# SpyDen: simplifying molecular and structural analysis across spines and dendrites

**DOI:** 10.1093/bioinformatics/btaf339

**Published:** 2025-06-16

**Authors:** Maximilian F Eggl, Surbhit Wagle, Jean P Filling, Thomas E Chater, Yukiko Goda, Tatjana Tchumatchenko

**Affiliations:** Institute of Experimental Epileptology and Cognition Research, University of Bonn Medical Centre, Bonn 53127, Germany; Institute of Neuroscience, CSIC-UMH, Alicante 03550, Spain; Institute of Experimental Epileptology and Cognition Research, University of Bonn Medical Centre, Bonn 53127, Germany; Institute of Physiological Chemistry, University of Mainz Medical Centre, Mainz 55128, Germany; Laboratory for Synaptic Plasticity and Connectivity, RIKEN Centre for Brain Science, Wako-Shi, Saitama 351-0198, Japan; Department of Physiology, Keio University School of Medicine, Shinjuku-ku, Tokyo 160-8582, Japan; Laboratory for Synaptic Plasticity and Connectivity, RIKEN Centre for Brain Science, Wako-Shi, Saitama 351-0198, Japan; Synapse Biology Unit, Okinawa Institute of Science and Technology Graduate University, Kunigami-gun, Okinawa 904-0495, Japan; Institute of Experimental Epileptology and Cognition Research, University of Bonn Medical Centre, Bonn 53127, Germany

## Abstract

**Motivation:**

Investigating the molecular composition of different neural compartments such as axons, dendrites, or synapses is critical for understanding learning and memory. State-of-the-art microscopy techniques now resolve individual molecules and pinpoint their position with a micrometer or nanometre resolution across hundreds of micrometres, allowing the labelling of multiple structures of interest simultaneously. Algorithmically, tracking individual molecules across hundreds of micrometres and determining whether they are inside a particular cellular compartment can be challenging. Historically, microscopy images are annotated manually, often using multiple software packages to detect fluorescence puncta and quantify cellular compartments of interest. Advanced ANN-based automated tools, while powerful, often can only help with selected parts of the data analysis, may be optimized for specific spatial resolutions, cell preparations, and may not be fully open source and open access to be sufficiently customizable.

**Results:**

Thus, we developed SpyDen, a Python package based upon three principles: (i) ease of use for multi-task scenarios, (ii) open-source accessibility and data export to a standard, open data format, (iii) the ability to edit any software-generated annotation and generalize across spatial resolutions. SpyDen operates on 2D microscopy time-series data, offering robust temporal tracking and spatial analysis capabilities. Equipped with a graphical user interface and accompanied by video tutorials, SpyDen provides a collection of powerful algorithms that can be used for neurite and synapse detection, fluorescent puncta, and intensity analysis. We validated SpyDen using expert annotation across numerous use cases to prove a powerful, integrated platform for efficient and reproducible molecular imaging analysis.

**Availability and implementation:**

SpyDen is available on https://github.com/meggl23/SpyDen while the compiled executables can be found at https://gin.g-node.org/CompNeuroNetworks/SpyDenTrainedNetwork.

## 1 Introduction

Neurons are highly morphologically diverse cells, with extensive projections, that typically range from micrometres (dendritic spines and axonal boutons) to mm’s/cm’s (dendrites and axons). The smallest neuronal synaptic sub-compartments contain molecules that are critical for the implementation of information processing in the brain (Abbott and Regehr 2004). Moreover, synaptic plasticity is mediated through a complex interaction of different molecular players, and is critical for learning and overall brain function (Yau 1976, Rosahl *et al.* 1993, Zilberter 2000, Yuste and Bonhoeffer 2001, Abbott and Regehr 2004, Chevaleyre *et al.* 2006, Lüscher and Malenka 2012, Frémaux and Gerstner 2015, Marblestone *et al.* 2016, Eggl *et al.* 2023, Wagle *et al.* 2023). Recent microscopy advances (Van Harreveld and Fifkova 1975, Desmond and Levy 1986, Hosokawa *et al.* 1995, Matsuzaki *et al.* 2004, Ding *et al.* 2009, Nagerl and Bonhoeffer 2010, Kwon and Sabatini 2011, Schermelleh *et al.* 2019, Padmanabhan *et al.* 2021) have enabled the study of the complex interactions between synaptic plasticity and neural function and the underlying molecular dynamics taking place over time across the dendritic tree, axons, and synapses at micrometer to nanometre scale. Additionally, these novel techniques allow for the quantification of the localization profiles of mRNAs and different protein species along the dendritic or axonal tree as well as within individual synapses, thereby providing insight into neuronal synaptic and dendritic dynamics at unprecedented resolution (Holt and Schuman 2013, Rangaraju *et al.* 2017, Helm *et al.* 2021). Common examples of challenges in analysing these datasets include (i) the reliance on manual annotation and subsequent segmentation, which can be time-consuming and laborintensive and (ii) the need to switch between tools to perform different analysis steps. The analysis can also be confounded by the observation that even experienced human annotators show a significant amount of variation (Graves *et al.* 2021, Fernholz *et al.* 2024). Additionally, the innate biological variability of neural anatomy (Mishchenko *et al.* 2010, Mukai *et al.* 2011, Berry and Nedivi 2017, Graves *et al.* 2021) and the challenges of tracking structures of interest across space and time (Fernholz *et al.* 2024) indicate the need for more quantitative and comprehensive analysis tools that can accommodate the diversity of cell compartments.

The increasing amount of high-resolution imaging data, coupled with the above-mentioned challenges, means that a more pragmatic approach to data analysis is to automate as much as is feasible (Ghani *et al.* 2017, Das *et al.* 2022). With the application of deep neural networks, the opportunity to perform automatic and reproducible analysis at a level similar to human-level annotation is within reach (Tong *et al.* 2021, Pchitskaya *et al.* 2023, Vogel *et al.* 2023). A common approach is to train artificial neural networks (often U-nets) to segment spines and dendrites in 3D from 2D stacked images and to generate 3D meshes (Ronneberger *et al.* 2015, Vidaurre-Gallart *et al.* 2022). Other artificial neural net approaches include the Matlab package *SpineS* (Argunşah *et al.* 2022), the DeepD3 Framework (Fernholz *et al.* 2024), or the work of Vogel *et al.* (2023). All these approaches represent powerful tools for the analysis of stretches of dendrite and offer the ability to utilize a pre-trained ANN or train a personal network.

However, several challenges can arise when applying these approaches. First, training deep learning models on custom datasets is time- and energy-consuming and requires machine learning knowledge. Second, the resulting segmentations can require further processing to extract data of interest, e.g. centres and ROIs of spines, distance of spines from soma or the medial axis path of the dendrite, or number of molecules of interest within a cell compartment (Iannella and Tanaka 2006, Kastellakis *et al.* 2015, Chater *et al.* 2022). Third, the resulting ROIs (dendrite and spines) are often not editable once calculated by the underlying algorithms. Providing a way to interact with the automatically generated results is critical for effective and robust analysis. This is particularly important if the underlying algorithm produces erroneous results in specific image areas. Fourth, some of the available tools require continuous, spatial signals, while others work with discrete puncta data and apply different definitions of cell compartments, making comparisons difficult. This makes a comprehensive and reproducible data analysis across different molecular data sets challenging.

In Table S4, available as supplementary data at *Bioinformatics* online, we provide a non-exhaustive list of available analysis tools and a comparative analysis of their respective features. This list includes tools that include at least a subset of the capabilities of SpyDen, i.e. to study any combination of dendrites, synapses, and mRNA and protein puncta. However, this means that in contrast to SpyDen, where this work can be done directly within one specific pipeline, e.g. analysing a dendritic stretch, the synaptic terminals along that stretch and then comparing protein puncta in each of that structure, certain tools lack features to perform this all-in-one analysis. For example, tools developed to segment dendrites and spines often lack features to quantify mRNA and protein puncta inside the segmented ROIs. Similarly, tools that can quantify fluorescent puncta require additional pre-processing to define ROIs. Hence, thus far, a more comprehensive analysis of neuronal images required switching between multiple tools and interfaces with various modus operandi, ranging from fully manual to fully automatic (Table S4, available as supplementary data at *Bioinformatics* online).

To close this gap and provide an all-in-one solution to both morphological and puncta image analysis, we present SpyDen, an open-access and open-source package to automatically evaluate and trace multiple features of interest, including dendrites and dendritic spines, as well as mRNA and protein localizations within those structures. Moreover, compared to existing tools, SpyDen outputs the measurements of interest in multiple human- and machine-readable format which can easily be used for further data analysis. This work includes a brief overview of the SpyDen workflow, a description of the underlying algorithms and a set of validations performed on a variety of datasets. Given this, we believe that SpyDen opens up new avenues for image analysis which are both time-efficient and reproducible in the study of dendrites and spines, as well as beyond into other cell types.

## 2 Materials and methods

### 2.1 Data analysis with SpyDen

We designed SpyDen as an integrated platform with the following properties:

- Reliable, cross-validated algorithms for the analysis of dendritic segments, somata, and dendritic spines that provide both the ROIs and relevant statistics across space and time that are bench-marked across different image resolutions using human expert performance.
- The integrated analysis of continuous molecular signals as well as discrete puncta that represent individual molecules across structures of interest means that comparisons between cell compartments and data types are possible within a single user interface and sets of parameters.
- A graphical user interface (see Fig. S1, available as supplementary data at *Bioinformatics* online) that can be used without any prior programming knowledge. By providing a compiled executable, scientists can utilize SpyDen without the need to read and write computer code. To further enhance the user experience, we provide video tutorials.
- SpyDen is fully open-source and open-access, containing no proprietary software. Its algorithms can be further customized to serve alternative analysis goals both within and outside the neuroscience context.
- All results from the automatic algorithms can be edited, both at the algorithm level and the level of local ROIs. This means that SpyDen provides a robust, algorithm-provided baseline that can be customized further if necessary.

**Figure 1.**
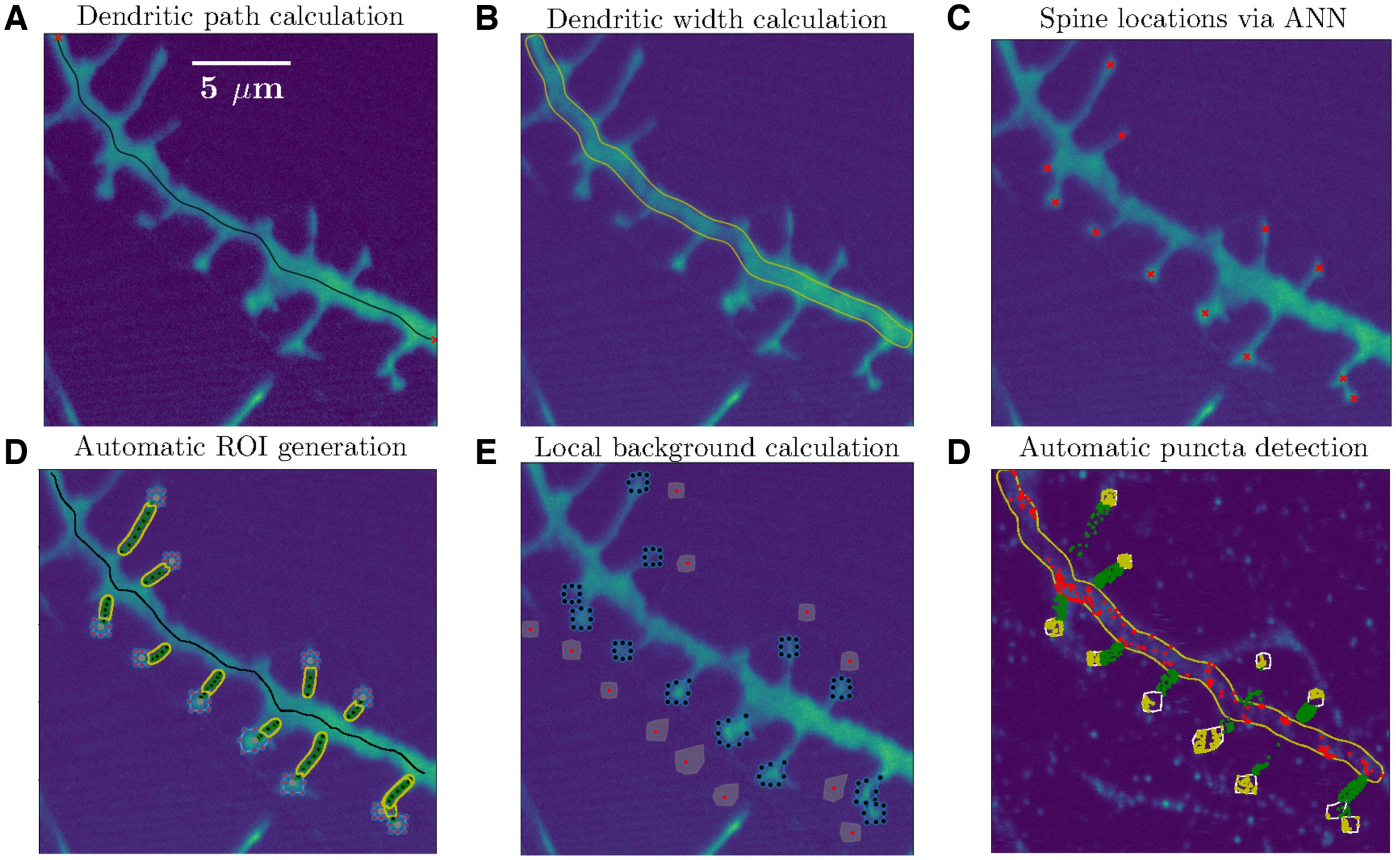
Steps of SpyDen’s data-analysis pipeline illustrated with an example experimental image. (A) Result of the medial axis calculation. The two red markers depict the user selection and the black line is the path through the dendrite connecting those points. (B) The width of the dendrite, using the medial axis, is calculated and depicted in yellow. (C) The crosses mark the spine locations, obtained here through the use of a neural network (manual selection can also be performed). (D) Using the spine locations, editable ROIs (depicted by the polygons with the red vertices) are calculated. Additionally, if a spine neck is visible in the image, its medial axis (black editable vertices) and ROI (yellow contours) are also generated. (E) To obtain statistics on the local background, the user can move the background location (depicted by the markers surrounded by the grey). (F) Using the dendritic ROI (red points, from *B*) and ROIs from the spines (yellow points, calculated in *D*) puncta can be calculated for fluorescence signal detection.

SpyDen combines a set of powerful algorithms within a single GUI that is optimized for ease of use across different analysis scenarios. This allows for efficient and reproducible analysis of neuronal images, avoiding the need to switch to different programs for different steps, reducing the number of potential errors, and speeding up image analysis. See Fig. 1 for an example of a complete workflow. Next, we detail the SpyDen workflow to demonstrate the above features and analysis steps, showing how SpyDen can be employed to tackle complex biological image analysis challenges and ensure precision and ease of use.

#### 2.1.1 Analysing dendritic segments

The first component in the SpyDen analysis pipeline is the generation of medial axis paths using user-provided start and end points that best describe a set of chosen dendrites. The resulting medial axis path (or paths) are available not only for dendrite analysis but can also be used for other parts of the analysis pipeline, e.g. spine analysis. An example of a medial axis path is shown in Fig. 1A, where the horizontal dendrite path has been generated (black line) connecting the two marked end points (red crosses).

**Figure 2.**
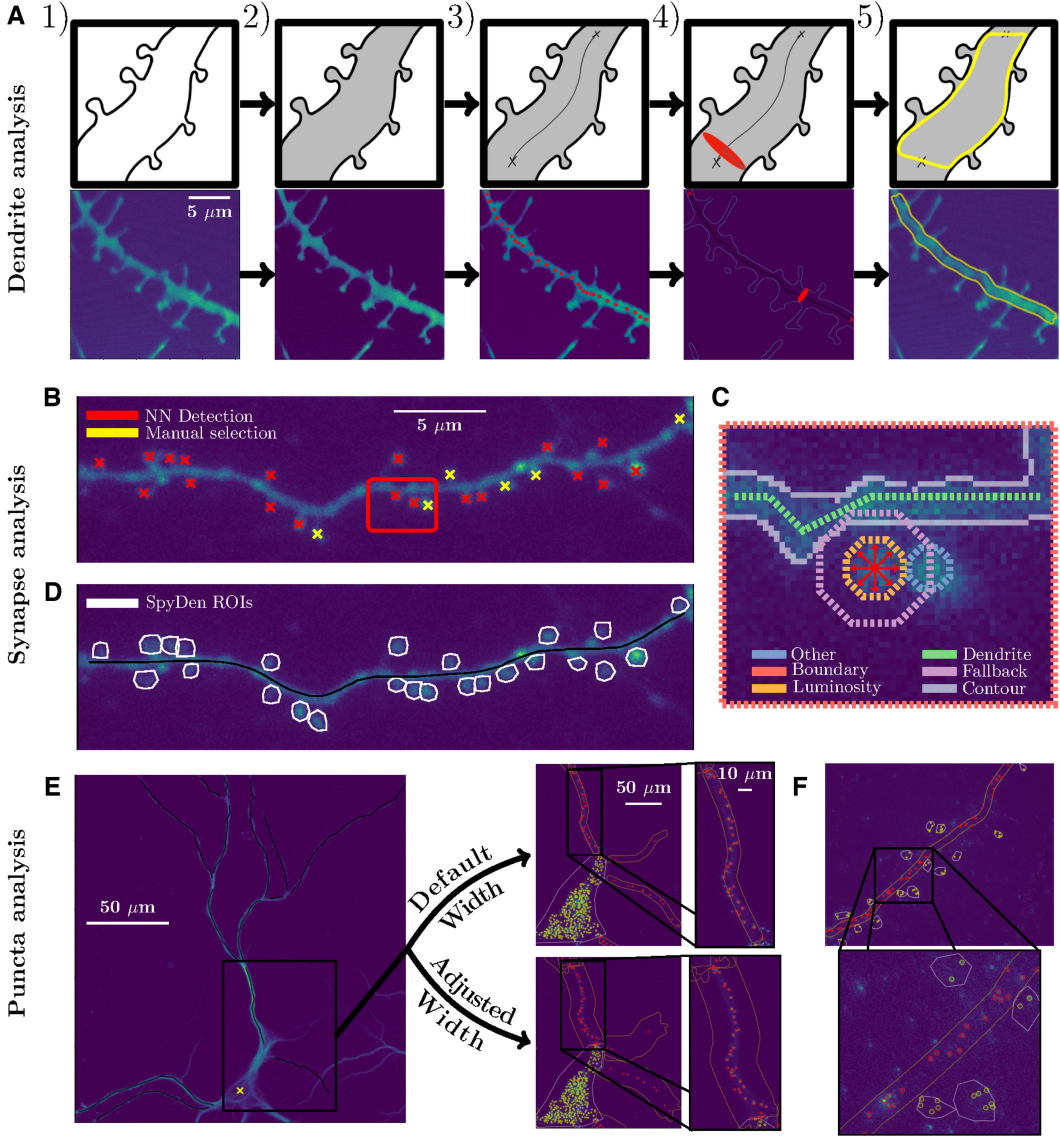
Illustrations of the three analysis pipelines of SpyDen with example images. (A) Analysis pipeline for generating the dendritic segmentation. From left to right: (1) Input image of the dendrite and the dendritic spines. (2) A median filter is applied to reduce the salt and pepper noise followed by a chosen threshold (default is the mean) for a rough segmentation of the fore- and background. (3) Given the start and end point of the dendrite, the medial axis path will be calculated. (4) For every point on the medial axis path we fit an ellipse that covers the dendrite to determine its width. (5) Applying a width increase condition, we require continuity in the dendritic width calculation and do not allow large abrupt changes that can occur due to the presence of spines. (B) An example dendritic stretch, where the spine heads have been marked manually (yellow) as well as using an artificial neural network (red). The box is additionally shown in a zoomed-in version in (C). (C) Using the selected centre of the spine (centre of the arrows), we project outwards in eight directions until a certain number of conditions are satisfied. The conditions are depicted with dashed lines in different colours on the plot. Example conditions are the dendrite rule (spine ROI should not intersect with the dendrite) or the fall-back rule (the spine ROI should exceed a certain threshold). (D) After iterating through all spine centres, and applying the heuristic rules to generate spine ROIs (polygons), we are left with a set of potential ROIs that can be further altered if necessary. (E and F) Once the ROIs (Soma, dendritic width—in E*—*and spine—in F) are finalized, puncta detection can be used to find bright spot puncta in the ROIs and get various measures for each punctum. Dendritic segmentation needs to be performed to obtain the dendritic puncta.

In line with our goal that all results arising from SpyDen algorithms should be editable, we provide the users the possibility to modify this path if desired (i) allowing for the selection of the channel that depicts the dendrite most reliably using the associated SpyDen sliders and (ii) including slider settings determining the level of background noise filtering. An example of noise filtering can be seen in Fig. 2A or for more details see Fig. S2, available as supplementary data at *Bioinformatics* online. While the above strategy allows for the global enhancement of the path, we also offer the users the ability to edit local parts of the path. Thus, we have included interactable vertices along the medial axis path (see Fig. 2A). These fully editable nodes (nodes can be moved, deleted, or new ones added) allow for fine adjustments to be made to single points of the path. In testing by experienced human annotators, GFP-filled dendrites with reasonable SNR required little to no adjustments.

With the medial axis path calculation results, SpyDen can then generate the full dendritic segmentation (see yellow outline in Fig. 1B). Once again, the result of this automatic process can be enhanced by using a set of sliders that alter the behaviour of the underlying segmentation algorithm. Examples of the segmentation results after altering these sliders can be seen in Fig. S2F–H, available as supplementary data at *Bioinformatics* online.

At this point, the results of the dendritic analysis can be saved in *.json* and *.csv* files (which are both human- and machine-readable). These files include statistics such as the width of the dendrite, luminosity along the medial axis and luminosity of the segmentation result (see Table S1, available as supplementary data at *Bioinformatics* online). The masks for dendritic segmentation for each dendrite are also saved as images. Additionally, the medial axis and dendrite segmentation ROIs are saved as *.png* and *.roi* files that either can be used for further post-processing or directly loaded into *ImageJ*, respectively. Finally, SpyDen allows users to import dendritic medial axis paths, obtained using other programs if they are in .npy and .roi format.

#### 2.1.2 Detecting and analysing dendritic spines

The typical density of dendritic spines along a dendrite in mammalian cortex is of the order of 1 spine/μm, meaning that even a relatively small part of an imaged neuron will contain tens to hundreds of spines. Even though it has been demonstrated that even experienced human annotators show significant variation when detecting and analysing spines (Graves *et al.* 2021, Fernholz *et al.* 2024), many experimental labs still rely on manual analysis, which is extremely time-consuming. To overcome this challenge and provide a baseline to reduce this variation, SpyDen combines automatic and manual approaches to provide spine statistics. On the one hand, a neural network-based implementation (see Fig. S4, available as supplementary data at *Bioinformatics* online, for a schematic) can be used for initial detection, which is then augmented by directly clicking on the spines (see Fig. 2B). Additional functionality is provided beyond merely selecting the spines: spine selections (both manual and automatic) can be deleted, and spines of particular interest (e.g. spines previously targeted for stimulation) can also be highlighted. This highlighting provides additional flexibility with regard to other cellular structures, such as labelled somata, that can be selected for analysis as if they were spines.

Given the set of spine locations, SpyDen then sequentially generates ROIs surrounding those structures. The ROI generation is based on a set of heuristic rules that take advantage of the inherent structure of the spines and rely on a set of parameters that can be tuned to achieve results that better fit the chosen experimental paradigm (for a depiction of these rules applied to an example spine, see Fig. 2C). Similarly, by using the dendritic medial axis algorithm, where the start point is the centre of the spine and the end point is where the spine intersects with the dendrite, we generate the spine neck path. This path is then segmented automatically to generate the spine neck ROI. We demonstrate the difference in ROI generation for both the spine head and neck when different parameters are chosen by interacting with the sliders provided by SpyDen in Fig. S5, available as supplementary data at *Bioinformatics* online. Similarly to the medial axis path generated for the dendritic branch, the spine ROIs and neck path are made up of interactable vertices (see Fig. 2D), which means that the automatic results can also be edited on a spine-to-spine level, if desired by the user.

SpyDen additionally provides a way to calculate the local background luminosity of each spine. Given that the local luminosity of the image surrounding the spine may impact its relative brightness and thus affect the resulting analysis, providing these background values is critical for accurate results. Therefore, SpyDen calculates a location close to each spine and uses the associated ROI to calculate the image’s local background luminosity (see Fig. 1E). Within the design philosophy of SpyDen, the location of this background can be manually adjusted if the algorithm chooses an unsuitable location.

A further challenge when analysing spines in temporal data is that the images may shift both on a global (on an image level if, e.g. the sample was disturbed) or on a local level (the innate microscopic movements of the spines or dendrites themselves). To combat this challenge, SpyDen provides both global and local motion correction and allows for accurate measurements. In the case of the local correction, manual intervention is once more provided by selecting a given timestep and dragging the associated ROI to the correct location.

Once all the above steps are completed, the spine statistics are stored as *.json* and *.csv* files and contain a wide variety of metrics for further post-processing. Additionally, *.png* and *.roi* files are generated of the spine polygons, which can be used for further analysis or figure generation.

#### 2.1.3 Detecting and analysing fluorescent puncta inside structures of interest

Finally, SpyDen takes advantage of its all-in-one nature to use the previous analysis (spatial image information) to detect and measure fluorescent puncta (discrete datapoints) inside the generated ROI. We consider three types of ROIs (polygonal spine ROIs, which also can be used to study soma and other similar structures, segmented line ROIs for dendrites, and segmented line ROIs for the spine necks), for each of these structures puncta are detected differently. For the spine polygonal ROIs and line ROIs of the spine necks, fluorescent puncta are detected inside the area enclosed by the polygon (yellow point and green points, respectively, Fig. 1F). For segmented line ROIs of the dendrites, the fluorescent puncta are detected by considering local rectangles defined by the cross-section and the closest line segments (red points, Fig. 1F). SpyDen provides the capability to filter out puncta of certain minimum and maximum sizes and omits puncta whose intensity is below the background noise level. This improves the efficiency of the algorithm and avoids detecting spurious puncta. The coordinates of the detected puncta are saved in human and machine-readable *.json* and *.csv* files.

### 2.2 SpyDen methods

#### 2.2.1 Algorithms for dendrite analysis

##### 2.2.1.1 Medial axis path calculation

To obtain the optimal medial axis that sits in the middle of the selected dendrite, we consider the treatment of the experimental image as an optimal path problem, i.e. given the start and end points provided, we attempt to find the best path through the dendrite. The image is transformed into a binary matrix, where the pixels that have luminosity over a given threshold represent an admissible path, and all other pixels are inaccessible (walls) (see Fig. S2B, available as supplementary data at *Bioinformatics* online). Given this formulation, standard pathing algorithms can be applied to obtain the shortest path calculation. SpyDen utilizes the standard Dijkstra algorithm of NetworkX (Hagberg *et al.* 2008) and speeds up the calculation time by downsampling the image if it is larger than 512 × 512 pixels.

However, if we solely applied the algorithm to the matrix described above, the path we generate would not lie within the middle of the dendrite but instead favour the dendrite edges (especially if the dendrite is curved or angled). Therefore, we augment the binary matrix, which represents the admissible dendrite pixels, with another factor that represents how far a given pixel is from the boundary of the dendrite. This factor is calculated by checking how far a given pixel is from an inadmissible pixel (a pixel below the luminosity threshold), i.e. a simple approximation of how far a given pixel is from the edge of the dendrite. This alteration ensures that the algorithm generates the shortest path at the dendrite’s centre. For an example of this augmented formulation, see Fig. S2C, available as supplementary data at *Bioinformatics* online, where the peaks represent the points furthest from the dendrite edge.

The resulting path consists of all pixels that connect the dendritic endpoints. Retaining all of these pixels, however, is inefficient, makes saving the dendrite for future use difficult, and complicates the editability of the dendritic path. To overcome these problems, the full-rank path is compressed by removing redundant pixels and generating a path consisting of a smaller set of control points. These control points, which define the editable nodes seen in Fig. 2A, are major turning points of the path and are obtained by a curvature-dependent sampling of the original path. By interpolating linearly between them, the full-rank path can be recovered. A pseudo-implementation of the above-described algorithm can be found in Algorithm S1, available as supplementary data at *Bioinformatics* online.

##### 2.2.1.2 Dendritic width calculation

Given the full-rank medial axis path, we can then proceed to segment the selected stretch of the dendrite in its entirety. To determine the exact width of the dendrite at each point, we define an ellipse at each pixel of the full-rank medial axis path. The semi-minor axis of these ellipses is set to a pre-set small value and points in the direction along the medial axis. In contrast, the semi-major axis is normal to the medial axis and is iteratively increased until it intersects with the boundary of the dendrite. An example illustration of an ellipse can be seen in the top panel of Fig. 2A. This dendrite boundary is calculated using the Canny edge detection algorithm (Canny 1986) applied to the median filtered and thresholded image. We employ this ellipse-based approach to generate the dendrite segmentation for two reasons: Firstly, directly using the canny edge detection detects all edges in the image, meaning that synaptic edges are not differentiated from the dendritic boundary. Secondly, depending on the luminosity profile of the image, certain edges may not be detected, leading to gaps in the dendritic boundary. By using ellipses and increasing their width iteratively, we are guaranteed to intersect with an edge at some point, which is not necessarily true if we solely used an outward-pointing ray. Examples of these phenomena can be seen in Fig. S3, available as supplementary data at *Bioinformatics* online.

To ensure that the dendritic width calculation does not include structures growing out of the dendrite (i.e. filopodia, spines, etc), the algorithm includes a smoothing condition that avoids abrupt changes in the width. To provide a means to interact with this algorithm, SpyDen includes two sliders that alter (i) the width-multiplication factor and (ii) the effect of the smoothing condition. These two sliders allow for significant alterations to the segmentation result, which in turn can enhance the result of the dendritic width calculation. The full algorithmic description of the dendritic width calculation can be seen in Algorithm S2, available as supplementary data at *Bioinformatics* online.

#### 2.2.2 Synaptic analysis

##### 2.2.2.1 Neural network-based identification of spine heads

Artificial neural networks are an effective tool to automatically annotate spines along dendritic stretches for further analysis (Ronneberger *et al.* 2015, Kashiwagi *et al.* 2019, Tong *et al.* 2021, Argunşah *et al.* 2022, Vogel *et al.* 2023, Fernholz *et al.* 2024). In most cases, this approach is employed directly for segmentation rather than the localization of spines along the dendritic branches. When spines are segmented directly, any additional ones not identified in the segmentation process can be difficult to include in later steps of the data-analysis pipeline. Additionally, most approaches mentioned above cannot edit and correct the generated ROIs once they are calculated.

These limitations mean that we opted to use a faster R-CNN architecture (Girshick 2015) to locate the centre of mass of spines directly [similar to the approach taken to Vogel *et al.* (2023)] rather than segmenting them. Concretely, the fast R-CNN architecture takes in an image of variable size and outputs a list of spine-bounding boxes and associated confidences. Given the centre of these boxes, we can then calculate the spine’s centre. Sets of spines from three different datasets (Helm *et al.* 2021, Chater *et al.* 2022, Fernholz *et al.* 2024) were manually labelled and used to train the neural network (pre-trained on ImageNet). Additional training images were generated by altering the original training set by changing their resolutions and rotating or mirroring them. For a set of example training images, as well as the schematic of the architecture, see Fig. S4, available as supplementary data at *Bioinformatics* online.

Focusing on locating spines, rather than segmenting them, maximizes intractability in SpyDen, as entries can easily be added or removed from the set of suggested spine centres. Additionally, the faster R-CNN architecture provides a confidence value for each identified spine that can be used to filter spines. Therefore, we have provided a slider in SpyDen that determines the minimum confidence the network must have for a given spine suggestion. The final detection can thus be a mix of automatic and manual spine locations to achieve the best result, as seen in Fig. 2B.

Throughout the development of SpyDen, we trained the underlying neural network on a wide variety of experimental image types, thus ensuring robustness across a wide variety of experiments (for details of the various experimental images used, see Table S5, available as supplementary data at *Bioinformatics* online). Nonetheless, we acknowledge that our network may not perform at the desired level for all images and note that this is still an open challenge in the field. However, inspired by the approach of DeepD3 (Fernholz *et al.* 2024), we include the ability not to use the default SpyDen network and instead provide a self-trained one that takes a 2D image as its input and generates a list of locations as its output (confidences can be arbitrarily set). By pressing the ‘Set NN (default)’ button (see box ii of Fig. S1, available as supplementary data at *Bioinformatics* online), SpyDen includes the option to download SpyDen’s default network or provides the path to a custom implementation. We emphasize that the neural network approach is optional for analysing the spines, and the fully manual procedure can also be utilized.

##### 2.2.2.2 Automatic ROI generation

As the above choice of spine identification algorithm only provides the spine centres, SpyDen necessarily includes a separate algorithm for spine segmentation. This algorithm takes the spine locations and then generates ROIs that can be used to analyse those spines.

Our approach relies on the inherent features of the spines to generate a polygon with editable vertices and provides three distinct advantages over common neural network-based approaches:

1. Using our approach leads to an ROI that consists of a polygon with editable vertices. Each vertex can be moved or deleted, and new vertices can be added to enhance the ROI, allowing for significant enhancement of the results once the ROIs are generated. The ROI generation algorithm parameters can also be adjusted to provide better automatic results.
2. By using a heuristic approach and relying on the spine features themselves, the algorithm is largely independent of image quality (unlike pre-trained neural network approaches), leading to a fundamentally robust algorithm across different experimental paradigms.
3. The rules that define the ROI generation are intuitive and can be tweaked, unlike the black-box approach of a pure neural network approach. Coupled with the open-source nature, this allows for altering the ROI generation algorithm of SpyDen as part of our inherent design goals.

The ROI generation then proceeds: a point interior to the spine, $s_{0}=(x_{0},y_{0})$, is supplied via the manual or automatic procedure. We then generate eight outward-pointing rays representing the cardinal and ordinal directions (N, S, E, W, and NW, SW, SE, and NE, respectively). Mathematically, we represent the *i*th point along each of these rays as


(1) $$s_{i,d}=s_{0}+i\times v_{d}$$

where $v_{d}$ is a given direction. An example of these rays can be seen in the red arrows of Fig. 2C. For each ray, we additionally introduce a counter $c_{d}$. This counter tracks how many times certain rules related to the spine morphology are broken as we step along the ray. When $c_{d}$ exceeds a certain value *n*, we halt the progression in this direction and denote this point as the edge of our ROI. The counter $c_{d}$ is independent for each ray, so the ROI generation will progress as long as one of the rays continues to increase.

Examples of the rules we use in SpyDen include the *luminosity drop-off rule* (when the value of the luminosity at point $s_{i,d}$ falls below a factor of the initial luminosity, $l_{0}$) or the *other spine rule* (when the point $s_{i,d}$ is closer to the centre of another spine than to its own spine centre). Examples of the locations where these rules are broken can be found plotted in different colours in Fig. 2C. The exact details of these rules can also be found in the Supplemental Material, available at *Bioinformatics* online.

Once the counters of all the rays have exceeded the value *n*, i.e. enough rules have been broken for all directions, we generate an octagonal shape encompassing the chosen spine, $s_{0}$, where the vertices are given by the final points along each ray. To increase the robustness of the algorithm, we then repeat this process by generating four additional polygons where the initial point is perturbed by one pixel in the cardinal directions. The resulting vertices of the five polygons are then averaged to generate a final polygon that has decreased dependence on the exact location of the initially provided spine position. The exact details of this algorithm can be seen in Algorithm S3, available as supplementary data at *Bioinformatics* online.

Finally, we comment on the difference between the ROI generation if *luminosity mode* or *area mode* is selected. Both these quantities are reliable proxies for the strength of a synaptic connection and have been used extensively to study synaptic plasticity (Matsuzaki *et al.* 2004, Hayashi-Takagi *et al.* 2015, Chater *et al.* 2022, Eggl *et al.* 2023). Nonetheless, care must be taken when generating the ROIs to study these quantities with temporal components. When studying the luminosity, generating an ROI encompassing the maximum extent of the spine over the entire period is critical. This allows for accurate measurement of the spine dynamics as the luminosity changes within the ROI. On the other hand, when studying the area, we need to generate an ROI that only encompasses the spine at *that* timepoint. Then, the areas of those ROIs dynamically change as the spine shrinks and grows. Therefore, in SpyDen, there is only one ROI in *luminosity mode* and an ROI for each snapshot in *area mode*. Please see Table S2, available as supplementary data at *Bioinformatics* online, for a detailed description of the output generated by the synaptic analysis in *luminosity mode* and *area mode*, respectively.

##### 2.2.2.3 Spine neck calculation

Relying on the same algorithms that allowed us to detect the dendritic medial axis and to trace the dendrite to provide the spine and dendrite location makes it possible to automate spine neck analysis and to customize it with user-provided slider settings. As the spine necks are much smaller than the dendritic stretches (which can cover the entire image), we only need to run these algorithms on a small part of the image where these structures are present, making the calculation significantly faster. Once the spine neck segmentation is complete, we remove any overlap with the spine head ROI for subsequent calculation.

We note that in certain cases it may not be possible to accurately identify the spine neck, because either it is not sufficiently illuminated or the spine itself is too close to the dendrite. In these cases, the user is notified that an attempt to generate the spine neck length was not successful and further segmentation of the neck was halted to avoid inaccurate results.

##### 2.2.2.4 Spine classification

Once the spine head and spine neck are computed, SpyDen uses their morphological features to classify each spine into one of the four classes: stubby, mushroom, thin, or outlier. To assign spine classes, we considered an adapted version of the fully automated, rule-based method provided in On *et al.* (2017). We use two variables: spine’s neck width (*NW*) and head width (*HW*). If the spine is missing a distinct neck region (i.e. NW=0), SpyDen classifies such spines as stubby. If the spine has a clear neck region (i.e. NW≠0), SpyDen calculates the ratio of neck width to head width ($\frac{NW}{HW}$). If this ratio is less than 0.5 (i.e. $\frac{NW}{HW}$ < 0.5), SpyDen classifies the spine as a mushroom spine. This is based on the morphology of mushroom spines that have a thick spine head and a relatively narrow neck. On the other hand, a thin spine lacks a clear spine head. Hence, SpyDen classifies the spine as thin if the ratio of neck width to spine width is between 0.5 and 1.1. Finally, if $\frac{NW}{HW}>1.1$, the spine is classified as an outlier. We provide the pseudo-code of our spine classification algorithm in Algorithm S4, available as supplementary data at *Bioinformatics* online.

#### 2.2.3 Motion correction

SpyDen also includes the capability of studying the temporal dynamics of dendrites and their spines. However, given that biological structures inevitably shift in time, be it through their innate motion or the noise of the experimental paradigm, it is critical to introduce a process to correct these shifts. Additionally, this movement may occur globally when the shifting is uniform for the entire image or on a local level, where individual sections may move independently from the rest of the image.

To account for these effects and accurately track the ROIs in time, we employ a phase cross-correlation approach (Foroosh *et al.* 2002, Guizar-Sicairos *et al.* 2008). Unlike spatial-domain algorithms, phase cross-correlation relies on the frequency representations of the images and, thus, is more robust to noise and occlusions. Furthermore, in comparison to directly calculating the cross-correlation of the two images, the spectral approach is significantly faster to calculate. This approach is particularly attractive because it can be applied to images of any size without significantly increasing computational time and can be used for global and local motion correction. Importantly, as the dynamic study of any biological system can include global motions (e.g. shifting of the entire image) as well as local motions (e.g. spine motility, plasticity, formation, and elimination), it is important to correct for these motions without masking the truly dynamics of the spines. To this end, SpyDen first performs a **global motion correction** step, which provides the necessary translations to account for the global shift which affects all structures in the image equally and then, if the user chooses to, performs a **local (spine-by-spine) motion correction** step. This latter step applies the phase cross-correlation to a small region around each spine and then instead of shifting the spine itself, shifts only the spine ROI to keep track of the spine’s relative motion. In doing so, we preserve the spine’s inherent changes in shape or position, ensuring that genuine structural alterations are not masked. Moreover, these local shifts are recorded in the final analysis file, allowing users to quantify and investigate spine motility over time.

In SpyDen, we find the necessary translations to account for the global shift and then introduce individual shift corrections via the same algorithm for each individual ROI. This algorithm is implemented by using skimage (van der Walt *et al.* 2014) to correct for both dendritic and synaptic shifting. Nonetheless, as the experimentally recorded spine movement may exceed the capabilities of the local shifting we implemented, we also introduced the ability to add a compensatory motion variable the spine ROIs at each timestep, the amount of this motion variable could be provided by a pre-registration step. The datasets we used to design our tool did not exhibit significant shifting but users interested in this feature can take advantage of the open-source code we provide and precompute the compensatory motion variable by registering the data sets using, e.g. SpineS Argunşah *et al.* (2022) or ImageJ Schneider *et al.* (2012) before importing these into SpyDen.

#### 2.2.4 Puncta analysis

Localizing and calculating the size of fluorescent bright puncta is a crucial component in the analysis performed on images following hybridization techniques such as fluorescent *in situ* hybridization (FISH) or single molecule FISH (smFISH). Such techniques result in a fluorescent bright puncta-like signal with a dark background (e.g. see Fig. 2E). Thus, given a dataset of multi-channel and/or multi-time images, SpyDen detects puncta in each of the provided images. This is achieved by using the Laplacian of Gaussian (LoG) technique (skimage implementation) to automatically detect fluorescent spots in the experimental images. This method requires a threshold value, defined as the absolute lower bound for scale space maxima. Nevertheless, the optimal threshold value may differ for distinct neurological structures. As SpyDen provides the ability to analyse fundamentally different biological structures (e.g. spines, dendrites, somata, etc.), we provide two sliders, labelled ‘Threshold dendrite’ and ‘Threshold synapse/soma’, respectively, to set the threshold values for each of the two types of ROIs independently. For example, given the image I(t,c,x,y) and an ROI, R(t,c,x,y), in image *I*, the threshold, $t_{R}(t,c)$, for *R* is determined as follows:


$$t_{R}(t,c)=\max(R(t,c,j,k))\times \frac{\gamma }{100}$$

Here, R(t,c,j,k) is the array containing fluorescent intensity value $\forall j,k\in {\text{ROI}}_{I}$ in channel *c* and at time *t* and γ represent the values of the time and channel sliders. Additionally, SpyDen includes the ability to detect puncta of various sizes by changing the minimum and maximum standard deviation of the Gaussian kernel using the provided puncta size slider. Finally, we omit puncta detection in ROIs whose intensity is below the background noise level to improve the efficiency of the algorithm and to avoid detecting erroneous puncta.

## 4 Results

As SpyDen consists of three fundamental analysis components (dendritic, synaptic, and puncta analysis), we perform three separate analyses to determine the effectiveness of using this tool. To this end, we take a variety of datasets that represent realistic experimental use-cases.

### 4.1 Dendritic analysis

**Figure 3.**
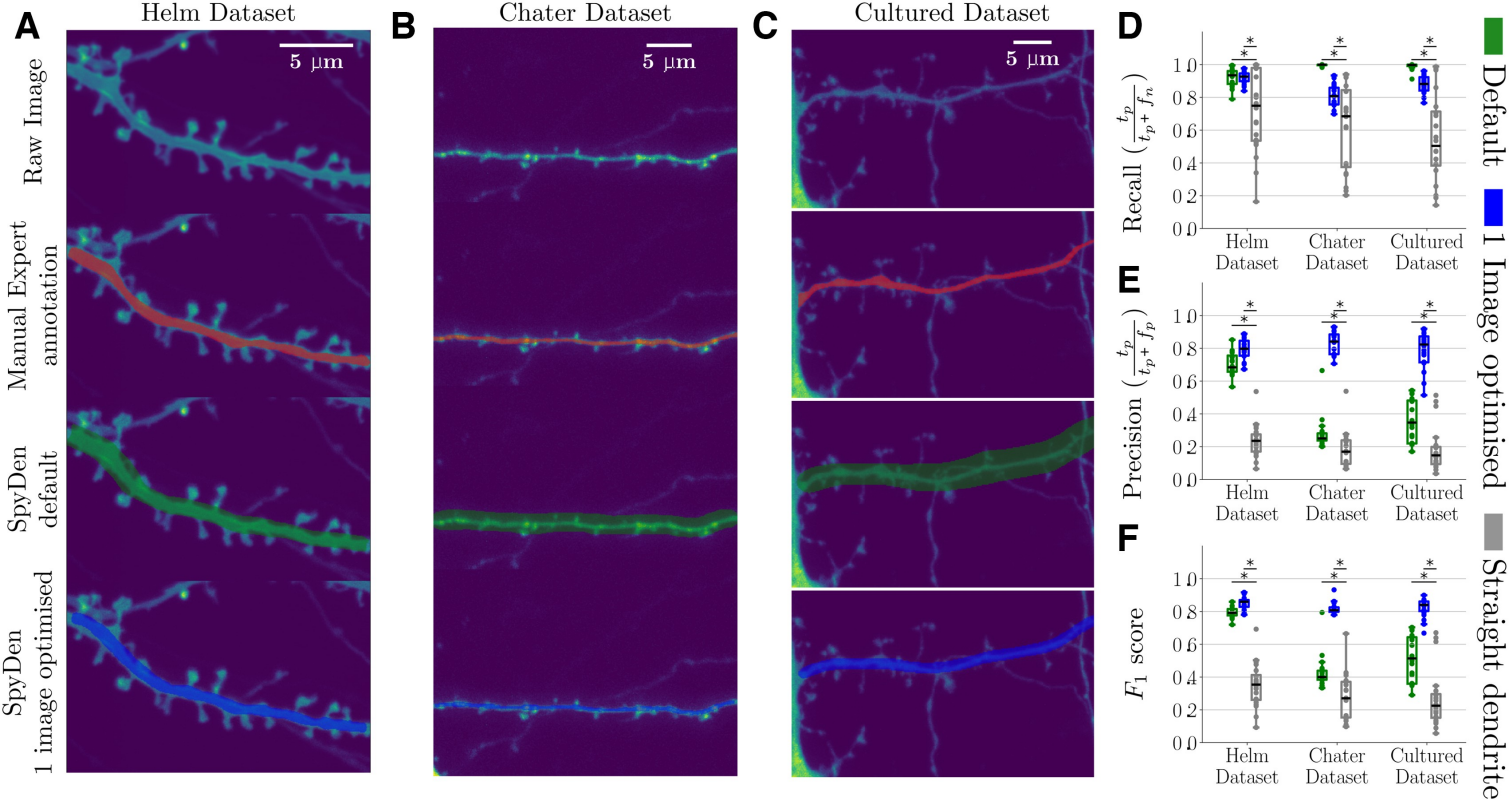
The dendritic segmentation in two different datasets reliably obtains performance comparable with manual expert evaluation. (A) Example dendrite segmentation of a dendrite from the Helm dataset (Helm *et al.* 2021). From top to bottom: Raw image, showing the dendritic stretch and associated spiny protrusions, manual annotation of the dendrite by an expert overlaid in red, segmentation achieved with the SpyDen tool if default values are used in green, and optimal segmentation if the algorithm parameters are augmented (blue). (B) As in (A), but using a set of data from the Chater dataset (Chater *et al.* 2022). Colour scheme remains the same. (C) As in (A), but using a set of data from the Cultured dataset. Colour scheme remains the same. (D–F) To measure the performance of dendritic segmentation, we use calculate the recall (D) (true positive results divided by the number of all samples that should have been identified as positive), precision (E) (number of true positive results divided by the number of all positive results), and $F_{1}$-score (F) which is a measure of our algorithms accuracy. In each figure, the green and blue refer to the performance of the SpyDen default and adapted parameters, respectively. Additionally, we calculated a baseline (called the *straight dendrite*), where a diagonal chord (2 μm width) is drawn between the dendritic start and end points and designated as the dendrite (grey). * refers to *P* < .05.

The synaptic and dendritic algorithms are evaluated on three distinct datasets to enhance the validity of the verification and prove the robustness of SpyDen to different experimental paradigms. These three datasets, publicly available and already published, are the Chater (Chater *et al.* 2022), Helm (Helm *et al.* 2021), and Cultured datasets (the last of which is unpublished and provided as part of this work). The first dataset contains examples of neurons from organotypic slice culture, while the latter two contain neurons from dissociated cultures, which makes the combination an attractive set of data for the verification of SpyDen. One key distinction between the datasets is the different experimental resolutions, a feature that many neural networks rely on to achieve their performance. Additionally, the difference in contrast between background noise and foreground means that conventional methods may struggle. For example, images from each of these datasets, see A, B, and C of Fig. 3. Nonetheless, we are aware that the SNR levels of our datasets are comparable, and so, to provide a robust evaluation of different experimental noise conditions, have performed an additional validation of the SpyDen algorithms by adding different levels of Gaussian noise to the image in Fig. S6, available as supplementary data at *Bioinformatics* online. Across all SNR levels, SpyDen was able to accurately segment the dendritic stretch. The synaptic analysis saw significantly more degradation in performance, but this intuitively follows from the fact that spines are disproportionately affected by noisy conditions. Nonetheless, up to a SNR ≈1, Spyden was able to reliably detect spines and generated ROIs.

We begin by studying the performance of the dendritic segmentation algorithm and compare this to the results of a manual expert evaluator. Each dataset comprises 20 dendrites. Natural use of SpyDen includes directly applying the results arising from the automatic algorithm, adjusting that result by modifying the medial axis path of the dendrite or by altering the algorithm parameter sliders. To mimic this natural use, we compare the expert to two distinct segmentations:

- Default: the automatic segmentation when the default parameters of SpyDen were used.
- One image optimized: the automatic segmentation resulting by altering the algorithm sliders akin to natural use for one image of the new dataset. These parameters are then used for all other images of that dataset.

Examples of dendrites from each dataset and the three different segmentations can be seen in Fig. 3, respectively.

Using the calculated segmentations, we can then generate metrics that quantify the performance of the SpyDen algorithm when compared to the ground truth (expert annotation). In our case, the segmentation task is equivalent to a classification task, as we are trying to classify each pixel as part of the dendrite. Additionally, this is an imbalanced task, as there are many more non-dendrite pixels than dendrite pixels. Metrics commonly used to evaluate the performance of classification algorithms in imbalanced datasets are the recall, precision, and $F_{1}$ scores (Powers 2020). Recall measures the ability of a model to correctly identify all relevant instances of the positive class (true positives) out of the total actual positive instances, precision measures the ability of a model to correctly identify positive instances without including too many false positives, and the $F_{1}$ score is the harmonic mean of precision and recall, defined as


(2) $$F_{1}=2\frac{\text{precision}\times \text{recall}}{\text{precision}+\text{recall}}$$

and provides a trade-off between these two metrics.

The results calculated for the two SpyDen segmentations can be seen across the Helm, Chater, and Cultured datasets in Fig. 3D–F. These figures also include the full equations of recall and precision. As each dataset has different characteristics, we expect to see variations in these metrics as the performance of SpyDen will naturally fluctuate. The algorithm annotations of the Helm dataset have high recall (≈0.9) and precision for both the default and altered parameters (≈0.8). This is at a level where there is little discernible difference between these two segmentations. We attribute this to the fact that the parameters of the dendrite algorithm were developed and tested with the experimental features of this dataset in mind. Therefore, altering the algorithm parameters to obtain a better segmentation leads to only a small improvement in the classification metrics. This leads to an average *F*1 score of 0.85, indicative of a well-performing model with a good balance between minimizing false positives and false negatives.

To further understand the performance of our model, we design a naive segmentation strategy that conceivably could generate reasonable dendritic segments, which we call the ‘straight dendrite’ (see grey lines points in Fig. 3D–F). This naive strategy is defined by taking the provided start and end points and drawing a chord of a certain thickness between these points. We found that the best average $F_{1}$ metric was achieved when this thickness was ≈2 μm, which aligns with experimentally reported dendritic widths (Stuart *et al.* 2016). Nevertheless, this baseline performs significantly lower than both the SpyDen segmentations and only achieves an average $F_{1}$ value of 0.4 and 0.3 for the Helm and Chater datasets, respectively. As the dendrites are not perfectly straight segments, this strategy is unable to deal with dendrites that may curve and thus deviate from the chord. This leads to low precision and highly variable recall.

Turning to the Chater dataset, we see that the default SpyDen parameters lead to a worse performance, which can be primarily attributed to the lower contrast between the background and the dendrite. Thus, the default parameters (particularly the variance of the Gaussian filter used to detect the edges of the dendrite) tend to overestimate the size of the dendrite, leading to a high recall rate (all points in the dendrite covered). However, this leads to a low precision rate as the segmentation includes many points of the background that are not part of the dendrite. Nonetheless, we still outperform the baseline strategy. With minimal adjustments to the algorithm parameters, we can achieve a performance comparable to that of the Helm Dataset ($F_{1}$ score ≈0.82). As SpyDen algorithm variables can be saved and transferred to other experimental images, the alteration of these parameters was only performed on one Chater dataset image and then applied to all other images. Thus, the dendrite segmentation of SpyDen can reliably obtain a satisfactory result across a wide array of experimental conditions. We note that the results of the Cultured dataset mirror those of the Chater dataset, i.e. the default SpyDen values represent an overestimation of the dendritic stretch, with high recall but low precision. Nonetheless, by augmenting the parameters to correct for this overestimation, we achieve a reasonable result with an $F_{1}$ score of ≈0.85. We note that with both approaches using SpyDen (automatic and augmented), the *F*1 performance is significantly better than the baseline.

We highlight that augmented segmentation was achieved without altering the path of the dendritic stretch (via the editable nodes). Therefore, an even higher $F_{1}$ score is possible. Nevertheless, as we aimed to mimic fast and efficient natural user analysis, we restricted ourselves to only changing the transferable parameters between different experimental images.

### 4.2 Spines

We now turn to verifying the abilities of SpyDen to accurately measure both the heads and necks of dendritic spines, using the previously employed datasets as they image both dendrites and spines. The ‘ground truth’ ROIs are obtained using manual annotation of the spines by expert evaluators, which are then compared against the results from SpyDen. As mentioned by Fernholz *et al.* (2024), using human expert annotators as the ground truth can be problematic as biases due to experience and experimental expertise can lead to different results. This means that comparing to only one annotator could lead to better or worse performance based on the results of that expert. To combat this, we used a set of three expert annotators that each worked on different datasets to hopefully provide the most ‘accurate’ ground truth. Here, the SpyDen head and neck ROIs are generated using the 1-image optimization approach mentioned above, i.e. we only change the parameters of the automatic algorithms once for each dataset and then leave them untouched. Nonetheless, we note that the results presented here could be further improved by taking advantage of SpyDen’s capability to change the ROIs directly.

**Figure 4.**
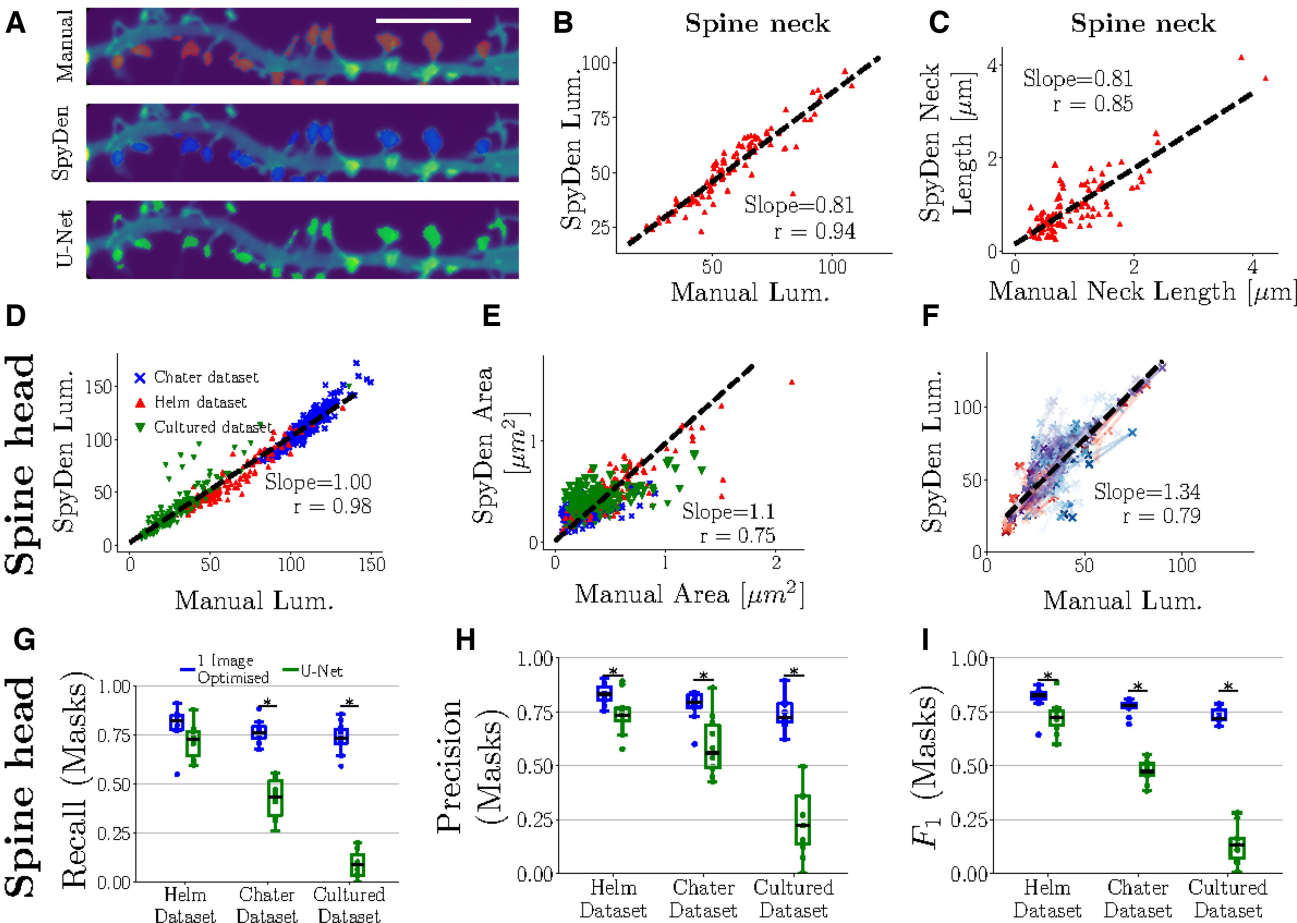
The synaptic detection and ROI generation of SpyDen achieves reliable performance when compared to a U-Net approach. (A) Example ROIs generated manually by an expert, the heuristic SpyDen rules, and a U-Net architecture. The white bar refers to 5 μm. (B and C) To compare the results obtained from the SpyDen analysis pipeline to annual expert annotation, we compare the average luminosity (B) and length of the SpyDen necks (C) against expert manual annotation. Here, we use the Helm *et al.* dataset. In both cases, we get good agreement with the manual expert evaluation. (D and E) To verify the results of the SpyDen analysis pipeline, we compare the average luminosity (D) and area (E) of the SpyDen ROIs against expert manual evaluation. We achieve robust agreement with the expert evaluation (for the three datasets), see superimposed linear fit. (F) Temporal data from three cells in the Chater dataset is used to evaluate the spine tracking capabilities of SpyDen. Each point represents an individual time point for a spine, with connecting lines illustrating the temporal evolution, and cells are distinguished by blue, purple, and red markers. We note that a good agreement is achieved between the luminosity calculated by SpyDen and that of the manual annotation of the expert. (G–I) We can directly compare these ROI masks to SpyDen’s performance. From left to right, we present the recall, precision, and *F*1 arising from SpyDen (blue) and the U-Net (green) while using the manual annotation as the ground truth. * refers to *P* < .05.

We compared metrics that describe the spine neck structure, i.e. the average luminosity of the spine neck (as evaluated by the ROI around the neck) and the neck length against the ground truth luminosity as evaluated manually in Fig. 4B and C, respectively. We found good agreement (Pearson correlation of 0.94 for luminosity and 0.85 for neck length) with the expert evaluation.

Similarly, we evaluated the SpyDen spine head ROIs and the luminosity of the spine head against manual annotation as both area and luminosity have been both used in the past (Matsuzaki *et al.* 2004, Hayashi-Takagi *et al.* 2015, Chater *et al.* 2022, Eggl *et al.* 2023) as proxies for synaptic strength (i.e. the number of AMPA receptors or PSD size). We find that the luminosities of the spines, as calculated by SpyDen, are in good agreement with those generated by the expert annotators (see Fig. 4A). When pooling all three datasets, the correlation exceeds 0.9, and the slope of the linear relationship between is close to unity. Additionally, SpyDen maintained this strong correlation when studying each dataset individually, albeit with slightly different linear relationships (see Fig. S7, available as supplementary data at *Bioinformatics* online, for individual linear fits). We also observed a correlation for areas (r=0.75). However, here the correlation between manual annotation and SpyDen performance was still high but lower than for luminosity. This could be due to the flexibility of manual annotation to use any possible shape while SpyDen parametrized all spine head ROIs as octagons.

Synapses are also dynamic structures that change constantly, a process known as synaptic plasticity. Therefore, we additionally need to verify that the algorithms underlying SpyDen can track these structures and provide accurate luminosity values over time. To this end, we took advantage of the temporal data provided by the Chater dataset and applied the automatic spine tracking provided by SpyDen. The results were then compared to those from an expert annotator (see Fig. 4F, where the different colours refer to three different analysed dendrites). We note that a good agreement is found between the two evaluations (*r* = 0.79). This good performance was achieved without any manual intervention to further enhance the results, which can be easily performed in SpyDen.

Next, we compare the ROI masks directly with those generated by the expert (as the luminosity can be obtained even without accurately covering the spine). Additionally, to see how our technique performs compared to other approaches, we trained a U-Net on the Helm dataset to output spine ROIs (Ronneberger *et al.* 2015). The U-Net takes the entire image as input and provides a binary mask of the same dimensions, highlighting possible spine areas. Given this structure, the U-Net might mark spines not part of the area we wish to analyse (e.g. lying on another dendritic branch). These extra selections would then unfairly decrease the accuracy of the U-Net, so all masks that the U-Net provided that lay outside the analysis region were removed. Nonetheless, we note that generating these extra (possibly erroneous) ROIs may be a problem that real users can encounter in real-world settings and that SpyDen does not have. Additionally, we note that the performance we would get by training the U-Net on all three of our test datasets would lead to trivially good results. However, training the network on this larger data set is computationally intense and not feasible for most end-users. Therefore, to mimic likely real-life analysis, we take the network and only train it on one dataset (in this case, the Helm dataset) and then apply it to all three datasets.

SpyDen, on the other hand, is not ‘trained’ on a given dataset or resolution and instead consists of deterministic algorithms that rely on the biological structure of the spines and, therefore, can generalize beyond the datasets we have used here. Additional generalization is achieved by enhancing the automatic results by altering the parameters underlying the algorithm and then applying these to all other images of the same experiment. This requires no knowledge of coding or computational resources, unlike retraining the neural network using the U-Net approach. We believe the above U-Net implementation represents the fairest comparison for SpyDen.

To evaluate the performance of the SpyDen and U-Net ROIs, we use the previously defined recall, precision, and $F_{1}$ metrics, using the expert annotation as the ‘ground truth’ baseline (Fig. 4G–I). Examples of these three ROIs can be seen in Fig. 4A, where red, green, and blue refer to the manual, SpyDen, and U-Net implementations, respectively. As expected, the U-Net and SpyDen perform at similar levels for the Helm dataset. However, SpyDen performs significantly better than the U-Net for the other two datasets, which follows intuitively as the U-Net is unable to adapt to the new experimental conditions. SpyDen achieves ≈0.8 for the three datasets, which is indicative of a well-performing model that does not rely on the image’s resolution to generate accurate results, making it a promising tool for evaluating spines across a wide array of datasets.

We also evaluated the performance of the SpyDen neural network in identifying spine centres in the three datasets (see Fig. S41, available as supplementary data at *Bioinformatics* online). We note that we obtain a consistent $F_{1}$ performance of ≈0.7 but emphasize that the spine centres provided by the neural network can be enhanced simply by adding or removing the suggested points with a single click, which cannot so simply done in the U-Net architecture.

### 4.3 Puncta

**Figure 5.**
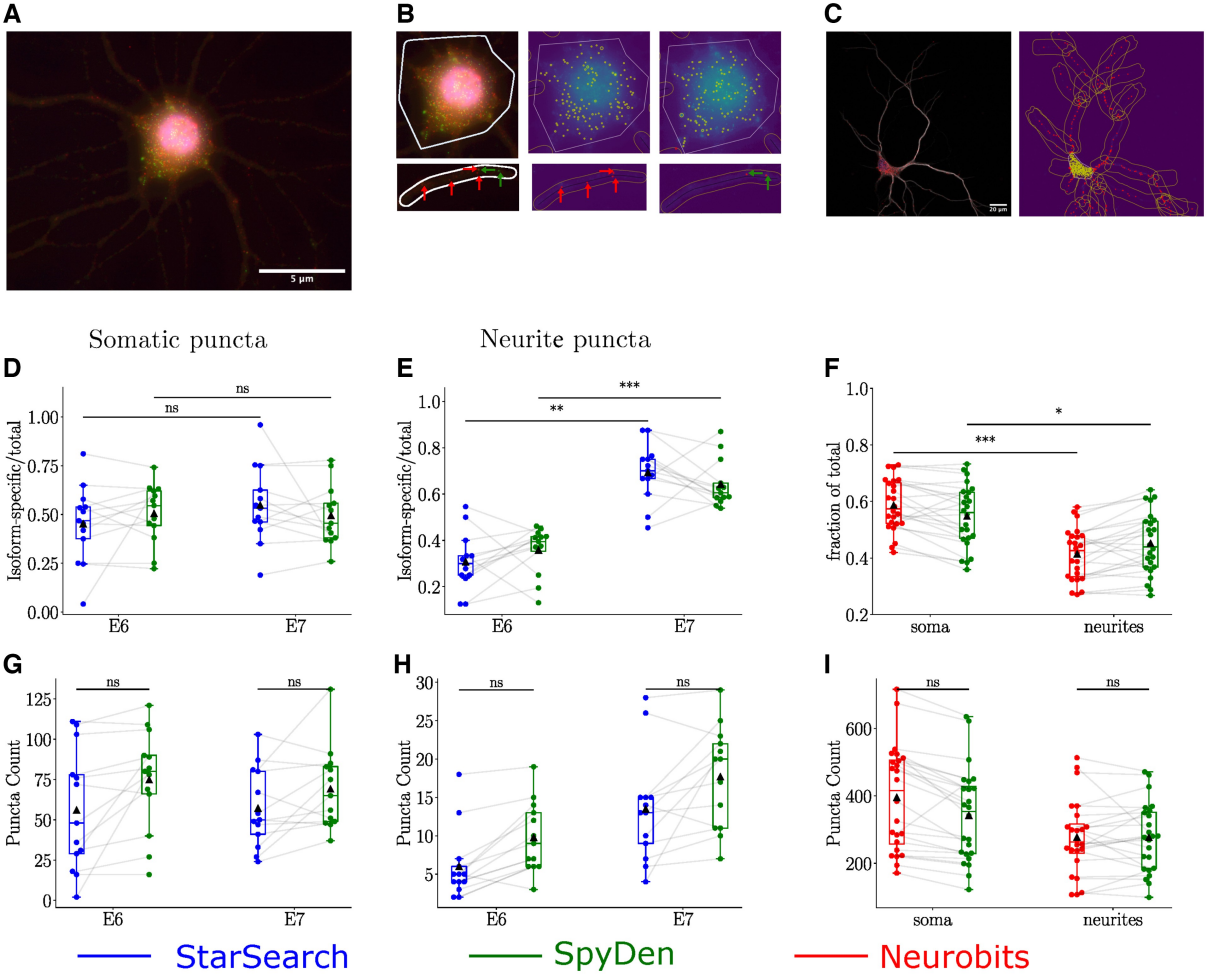
Validation of SpyDen’s puncta detection pipeline on published datasets. (A) smFISH of Cddc42E6 (green channel) and Cdc42E7 isoforms (red channel), cell body is labelled with DAPI (blue channel). Arrows point to individual puncta. (B) Zoomed-in image of the segmented soma and an example dendrite with smFISH puncta for both isoforms (E6 in green, E7 in red) on the left, same puncta as detected by SpyDen on right. (C) FISH of CaMKIIα mRNA in red with fluorescent immunolabeling of MAP2 for neurites (on left) and corresponding somatic, dendritic, and identified mRNA puncta by SpyDen on the right. (D) The ratio of isoform specific relative to total Cdc42 in somata based on puncta count by StarSearch and SpyDen. Ratios based on results from SpyDen are comparable to those of StarSearch. (E) Same analysis as in (D) for neurites. Isoform E7 has statistically higher localization than E6. The same is obtained from the results of SpyDen analysis (*: *P*-value <.05,**: *P*-value <.01, ***: *P*-value <.001). (F) Soma vs neurites fraction of total CaMKIIα mRNA with higher localization in soma. The analysis using SpyDen is able to replicate the results obtained using Neurobits. (G–I) Comparison of smFISH puncta count by StarSearch vs SpyDen in soma (G), in neurites (H), and FISH puncta count by Neurobits vs SpyDen (I).

To test the accuracy and efficiency of the SpyDen puncta analysis, we compared the results of SpyDen on two recently published data sets (Ciolli Mattioli *et al.* 2019, Fonkeu *et al.* 2019). The first dataset consists of images of mouse primary cortical neurons, in which two different Cdc42 isoforms were labelled using single molecule mRNA FISH (shown in Fig. 5A). Dataset2 contains images of rat dissociated hippocampal cultured neurons, on which mRNA FISH was performed for CaMKIIα (shown in Fig. 5C, left). In the original work, quantification of smFISH puncta for both of Cdc42 isoforms (namely, E6 and E7) was performed using StarSearch (RajLab), which is a well known and frequently used tool for smFISH puncta quantification. The results from SpyDen’s analysis were comparable to those from the original work in terms of the percentage localization of the two isoforms between soma and neurites (see Fig. 5D and E). Similarly, in the original work, dataset 2 was analysed using Neurobits (Tushev *et al.* 2018). Again, the quantification of CaMKIIα mRNA from SpyDen in somatic and dendritic compartments matched well with the results obtained using *Neurobits* (see Fig. 5F). Furthermore, we obtained comparable results in terms of the count of puncta detected in each compartment (soma vs neurites) in both data sets (see Fig. 5G, H, and I and Fig. S8, available as supplementary data at *Bioinformatics* online). One of the advantages of using SpyDen puncta detection is that it allows manual enhancements by providing the option to inspect and alter the underlying algorithm parameters (such as threshold values or puncta size). Additionally, SpyDen’s approach promotes efficient evaluation of the puncta; instead of identifying puncta in the whole image (as is done in *Neurobits*), SpyDen looks for puncta only in designated ROIs. This greatly reduces the time spent analysing each image. Another advantage of using the SpyDen puncta detection is that we provide video tutorials demonstrating how to use the analysis pipeline. This means that learning how to use SpyDen is much easier than learning how to use other tools that only provide text-based documentation. Moreover, existing tools often do not provide useful statistics which are automatically output by SpyDen, such as the size of each punctum, their location, and minimum and maximum intensity. For a complete list of puncta statistics, see Table S3, available as supplementary data at *Bioinformatics* online.

## 5 Discussion

With SpyDen, we have presented a robust and versatile tool for studying continuous and discrete molecular localizations inside dendrites and dendritic spine compartments. While we developed SpyDen with dendritic compartments in mind, the algorithms underlying SpyDen are also readily applicable to any other cellular compartments, e.g. fluorescently labelled nuclei within an area of interest or the number of molecules of interest inside a specific cellular compartment. To aid the adaptation and ease of use, we have incorporated into SpyDen three fundamental principles: (i) easy-to-use and intuitive operation without prior knowledge of programming due to a graphical user interface and video tutorials, (ii) it is fully open-source and open-access, containing no proprietary software, (iii) results arising from the ANN-based tracing and ROI algorithms are editable. In addition, with SpyDen, a more comprehensive analysis of multi-channel, time-series neuronal images, can be performed which none of the existing tools provide solely (see Table S4, available as supplementary data at *Bioinformatics* online).

Finally, we cross-validated and bench-marked SpyDen performance across different experimental datasets and have shown that SpyDen achieves reliable results when asked to trace dendrites, identify spines, and fluorescent puncta and its performance was on par with expert manual annotators. On top of the built-in algorithm-based solution, manual curation of ROIs as well as other objects of interest can further enhance the analysis because each step of the SpyDen pipeline can be user-adapted, even after a parameter reference point was suggested by built-in algorithms or imported from previous analyses.

SpyDen was designed as a one-stop option for a full data-analysis pipeline that provides a GUI for users without coding experience to take advantage of powerful algorithms to enhance their analysis. Nonetheless, for dendritic segmentation, SpyDen requires a certain amount of user interaction, whether by providing the dendritic end points, adjusting slider settings, or editing spine ROIs. We emphasize that the open-source nature of our tool means that users can adapt our algorithms so that, e.g. candidate dendritic end points that serve their needs are suggested automatically, and thus be more amenable to large-scale datasets. Additionally, while SpyDen does not currently parallelize or upscale the detection process in a fully automated manner—partly due to the need for user-informed steps, we believe providing a robust, integratable framework is an important first step. This approach alleviates the need for multiple specialized software solutions, thereby streamlining the workflow and encouraging further community-driven innovations to enable more advanced automation and parallelization in the future. However, future work will focus on extending SpyDen in such a way that the analysis of large datasets can also be handled robustly and efficiently.

An important design choice in SpyDen was opting for a heuristic-based approach rather than relying on artificial neural networks for the dendritic and synapse segmentation as well as the puncta analysis. While previous studies (e.g. Argunşah *et al.* (2022) or Fernholz *et al.* (2024)) have demonstrated the power of ANNs for such tasks, these methods are often ‘black boxes’ that do not give much insight into their decision making process. Instead, we aimed to provide a different approach that aims to be transparent, easily customizable, and more readily adapted to user-specific experimental contexts. Although this choice necessarily sacrifices some of the potential automation and speed of ANN-based solutions, it provides a flexible platform that users can fine-tune—both at the code level and via adjustable parameters. Nonetheless, with the steadily increasing capabilities of ANNs, future iterations of SpyDen will aim to incorporate these technologies to enhance its analysis pipeline.

In summary, SpyDen provides a powerful open-access and open-code platform for cellular studies across different cellular compartments, discrete as well as continuous signals of interest and different image resolutions.

## Supplementary Material

btaf339_Supplementary_Data

## Data Availability

Data and code to generate the figures found in this article can be found in the following public GitHub repository SpydenPaperFigs. The full code base for SpyDen, including instructions on installation and use, can be found at the github repository SpyDen while the compiled executables can be found at gin.g-node.org/CompNeuroNetworks/SpyDenTrainedNetwork.

## Statistical methods

Comparisons between expert manual and SpyDen results were performed using a Studentized *t*-test and multiple comparisons were corrected for using a Bonferroni correction factor.

## References

[btaf339-B1] Abbott LF , Regehr WG. Synaptic computation. Nature 2004;431:796–803. 15483601 10.1038/nature03010

[btaf339-B2] Argunşah AO , Erdil E, Ghani MU et al An interactive time series image analysis software for dendritic spines. Sci Rep 2022;12:12405. 35859092 10.1038/s41598-022-16137-y PMC9300710

[btaf339-B3] Berry KP , Nedivi E. Spine dynamics: are they all the same? Neuron 2017;96:43–55. 28957675 10.1016/j.neuron.2017.08.008 PMC5661952

[btaf339-B4] Canny J. A computational approach to edge detection. IEEE Trans Pattern Anal Mach Intell 1986;8:679–98. 21869365

[btaf339-B5] Cauzzo S , Bruno E, Boulet D et al A modular framework for multi-scale tissue imaging and neuronal segmentation. Nat Commun 2024;15:4102. 38778027 10.1038/s41467-024-48146-y PMC11111705

[btaf339-B6] Chater T , Eggl M, Goda Y et al A quantitative rule to explain multi-spine plasticity. bioRxiv, 2022–07, 10.1101/2022.07.04.498706, 2022, preprint: not peer reviewed.

[btaf339-B7] Chevaleyre V , Takahashi KA, Castillo PE. Endocannabinoid-mediated synaptic plasticity in the CNS. Annu Rev Neurosci 2006;29:37–76. 16776579 10.1146/annurev.neuro.29.051605.112834

[btaf339-B8] Ciolli Mattioli C , Rom A, Franke V et al Alternative 3 UTRs direct localization of functionally diverse protein isoforms in neuronal compartments. Nucleic Acids Res 2019;47:2560–73. 30590745 10.1093/nar/gky1270 PMC6411841

[btaf339-B9] Danielson E , Lee SH. SynPAnal: software for rapid quantification of the density and intensity of protein puncta from fluorescence microscopy images of neurons. PLoS One 2014;9:e115298. 25531531 10.1371/journal.pone.0115298 PMC4274056

[btaf339-B10] Das N , Baczynska E, Bijata M et al 3dSpAn: an interactive software for 3D segmentation and analysis of dendritic spines. Neuroinformatics 2022;20:679–98. 34743262 10.1007/s12021-021-09549-0

[btaf339-B11] Desmond NL , Levy WB. Changes in the numerical density of synaptic contacts with long-term potentiation in the hippocampal dentate gyrus. J Comp Neurol 1986;253:466–75. 3025272 10.1002/cne.902530404

[btaf339-B12] Ding JB , Takasaki KT, Sabatini BL. Supraresolution imaging in brain slices using stimulated-emission depletion two-photon laser scanning microscopy. Neuron 2009;63:429–37. 19709626 10.1016/j.neuron.2009.07.011 PMC2756148

[btaf339-B13] Eggl MF , Chater TE, Petkovic J et al Linking spontaneous and stimulated spine dynamics. Commun Biol 2023;6:930. 37696988 10.1038/s42003-023-05303-1 PMC10495434

[btaf339-B14] Pchitskaya E , Vasiliev P, Smirnova D et al SpineTool is an open-source software for analysis of morphology of dendritic spines. Sci Rep 2023;13:10561. 37386071 10.1038/s41598-023-37406-4 PMC10310755

[btaf339-B15] Fernholz MHP , Guggiana Nilo DA, Bonhoeffer T et al DeepD3, an open framework for automated quantification of dendritic spines. PLoS Comput Biol 2024;20:e1011774. 38422112 10.1371/journal.pcbi.1011774 PMC10903918

[btaf339-B16] Fonkeu Y , Kraynyukova N, Hafner A-S et al How mRNA localization and protein synthesis sites influence dendritic protein distribution and dynamics. Neuron 2019;103:1109–22.e7. 31350097 10.1016/j.neuron.2019.06.022

[btaf339-B17] Foroosh H , Zerubia JB, Berthod M. Extension of phase correlation to subpixel registration. IEEE Trans Image Process 2002;11:188–200. 18244623 10.1109/83.988953

[btaf339-B18] Frémaux N , Gerstner W. Neuromodulated spike-timing-dependent plasticity, and theory of three-factor learning rules. Front Neural Circuits 2015;9:85. 26834568 10.3389/fncir.2015.00085 PMC4717313

[btaf339-B19] Garcia SB , Schlotter AP, Pereira D et al RESPAN: an accurate, unbiased and automated pipeline for analysis of dendritic morphology and dendritic spine mapping. bioRxiv, 2024–06, 10.1101/2024.06.06.597812, 2024, preprint: not peer reviewed.

[btaf339-B20] Ghani MU , Mesadi F, Kanık SD et al Dendritic spine classification using shape and appearance features based on two-photon microscopy. J Neurosci Methods 2017;279:13–21. 27998713 10.1016/j.jneumeth.2016.12.006

[btaf339-B21] Gilles J-F , Mailly P, Ferreira T et al Spot Spine, a freely available ImageJ plugin for 3D detection and morphological analysis of dendritic spines. F1000Res 2024;13:176. 39318716 10.12688/f1000research.146327.2 PMC11420623

[btaf339-B22] Girshick R. Fast R-CNN. In: *Proceedings of the IEEE International Conference on Computer Vision*. New York City, NY (United States): IEEE, 2015, 1440–8.

[btaf339-B23] Graves AR , Roth RH, Tan HL, et al Visualizing synaptic plasticity in vivo by large-scale imaging of endogenous AMPA receptors. Elife 2021;10:e66809. 34658338 10.7554/eLife.66809 PMC8616579

[btaf339-B24] Guizar-Sicairos M , Thurman ST, Fienup JR. Efficient subpixel image registration algorithms. Opt Lett 2008;33:156–8. 18197224 10.1364/ol.33.000156

[btaf339-B25] Hagberg A , Swart P, Chult DS. Exploring network structure, dynamics, and function using networkx. Technical report. Los Alamos National Lab (LANL), Los Alamos, NM (United States), 2008.

[btaf339-B26] Hayashi-Takagi A , Yagishita S, Nakamura M et al Labelling and optical erasure of synaptic memory traces in the motor cortex. Nature 2015;525:333–8. 26352471 10.1038/nature15257 PMC4634641

[btaf339-B27] Helm MS , Dankovich TM, Mandad S et al A large-scale nanoscopy and biochemistry analysis of postsynaptic dendritic spines. Nat Neurosci 2021;24:1151–62. 34168338 10.1038/s41593-021-00874-w

[btaf339-B28] Holt CE , Schuman EM. The central dogma decentralized: new perspectives on RNA function and local translation in neurons. Neuron 2013;80:648–57. 24183017 10.1016/j.neuron.2013.10.036 PMC3820025

[btaf339-B29] Hosokawa T , Rusakov DA, Bliss TV et al Repeated confocal imaging of individual dendritic spines in the living hippocampal slice: evidence for changes in length and orientation associated with chemically induced LTP. J Neurosci 1995;15:5560–73. 7643201 10.1523/JNEUROSCI.15-08-05560.1995 PMC6577640

[btaf339-B30] Iannella N , Tanaka S. Synaptic efficacy cluster formation across the dendrite via STDP. Neurosci Lett 2006;403:24–9. 16762502 10.1016/j.neulet.2006.03.079

[btaf339-B31] Kashiwagi Y , Higashi T, Obashi K et al Computational geometry analysis of dendritic spines by structured illumination microscopy. Nat Commun 2019;10:1285. 30894537 10.1038/s41467-019-09337-0 PMC6427002

[btaf339-B32] Kastellakis G , Cai DJ, Mednick SC et al Synaptic clustering within dendrites: an emerging theory of memory formation. Prog Neurobiol 2015;126:19–35. 25576663 10.1016/j.pneurobio.2014.12.002 PMC4361279

[btaf339-B33] Kwon H-B , Sabatini BL. Glutamate induces de novo growth of functional spines in developing cortex. Nature 2011;474:100–4. 21552280 10.1038/nature09986 PMC3107907

[btaf339-B34] Levet F , Tønnesen J, Nägerl UV et al SpineJ: a software tool for quantitative analysis of nanoscale spine morphology. Methods 2020;174:49–55. 32006677 10.1016/j.ymeth.2020.01.020

[btaf339-B35] Lüscher C , Malenka RC. NMDA receptor-dependent long-term potentiation and long-term depression (LTP/LTD). Cold Spring Harb Perspect Biol 2012;4:a005710. 22510460 10.1101/cshperspect.a005710 PMC3367554

[btaf339-B36] Marblestone AH , Wayne G, Kording KP. Toward an integration of deep learning and neuroscience. Front Comput Neurosci 2016;10:94. 27683554 10.3389/fncom.2016.00094 PMC5021692

[btaf339-B37] Matsuzaki M , Honkura N, Ellis-Davies GCR et al Structural basis of long-term potentiation in single dendritic spines. Nature 2004;429:761–6. 15190253 10.1038/nature02617 PMC4158816

[btaf339-B38] Mishchenko Y , Hu T, Spacek J et al Ultrastructural analysis of hippocampal neuropil from the connectomics perspective. Neuron 2010;67:1009–20. 20869597 10.1016/j.neuron.2010.08.014 PMC3215280

[btaf339-B39] Mukai H , Hatanaka Y, Mitsuhashi K et al Automated analysis of spines from confocal laser microscopy images: application to the discrimination of androgen and estrogen effects on spinogenesis. Cerebral Cortex 2011;21:2704–11. 21527787 10.1093/cercor/bhr059 PMC3209797

[btaf339-B40] Nagerl UV , Bonhoeffer T. Imaging living synapses at the nanoscale by STED microscopy. J Neurosci 2010;30:9341–6. 20631162 10.1523/JNEUROSCI.0990-10.2010 PMC6632439

[btaf339-B41] On V , Zahedi A, Ethell IM et al Automated spatio-temporal analysis of dendritic spines and related protein dynamics. PLoS One 2017;12:e0182958. 28827828 10.1371/journal.pone.0182958 PMC5565271

[btaf339-B42] Padmanabhan P , Kneynsberg A, Götz J. Super-resolution microscopy: a closer look at synaptic dysfunction in Alzheimer disease. Nat Rev Neurosci 2021;22:723–40. 34725519 10.1038/s41583-021-00531-y

[btaf339-B43] Powers DMW. Evaluation: from precision, recall and F-measure to ROC, informedness, markedness and correlation. J Mach Learn Technol 2011;2:37.

[btaf339-B44] Rangaraju V , Tom Dieck S, Schuman EM. Local translation in neuronal compartments: how local is local? EMBO Rep 2017;18:693–711. 28404606 10.15252/embr.201744045 PMC5412868

[btaf339-B45] Ronneberger O , Fischer P, Brox T. U-Net: convolutional networks for biomedical image segmentation. In: *Proceedings: Medical Image Computing and Computer-Assisted Intervention*. MICCAI, Minnesota (MN), 2015, 234–41.

[btaf339-B46] Rosahl TW , Geppert M, Spillane D et al Short-term synaptic plasticity is altered in mice lacking synapsin I. Cell 1993;75:661–70. 7902212 10.1016/0092-8674(93)90487-b

[btaf339-B47] Savage JT , Ramirez JJ, Risher WC et al SynBot is an open-source image analysis software for automated quantification of synapses. Cell Rep Methods 2024;4:100861. 39255792 10.1016/j.crmeth.2024.100861 PMC11440803

[btaf339-B48] Schermelleh L , Ferrand A, Huser T et al Super-resolution microscopy demystified. Nat Cell Biol 2019;21:72–84. 30602772 10.1038/s41556-018-0251-8

[btaf339-B49] Schmied C , Soykan T, Bolz S et al SynActJ: easy-to-use automated analysis of synaptic activity. Front Comput Sci 2021;3:777837.

[btaf339-B50] Schneider CA , Rasband WS, Eliceiri KW. NIH image to ImageJ: 25 years of image analysis. Nat Methods 2012;9:671–5. 22930834 10.1038/nmeth.2089 PMC5554542

[btaf339-B51] Shah SI , Ong HL, Demuro A et al PunctaSpecks: a tool for automated detection, tracking, and analysis of multiple types of fluorescently labeled biomolecules. Cell Calcium 2020;89:102224. 32502904 10.1016/j.ceca.2020.102224 PMC7343294

[btaf339-B52] Stuart G , Spruston N, Häusser M. Dendrites. Oxford, UK: Oxford University Press, 2016.

[btaf339-B53] Tong L , Langton R, Glykys J et al ANMAF: an automated neuronal morphology analysis framework using convolutional neural networks. Sci Rep 2021;11:8179. 33854113 10.1038/s41598-021-87471-w PMC8046969

[btaf339-B54] Tushev G , Glock C, Heumüller M et al Alternative 3 UTRs modify the localization, regulatory potential, stability, and plasticity of mRNAs in neuronal compartments. Neuron 2018;98:495–511.e6. 29656876 10.1016/j.neuron.2018.03.030

[btaf339-B55] van der Walt S , Schönberger JL, Nunez-Iglesias J et al; scikit-image contributors. scikit-image: image processing in python. PeerJ 2014;2:e453. 25024921 10.7717/peerj.453 PMC4081273

[btaf339-B56] Van Harreveld A , Fifkova E. Swelling of dendritic spines in the fascia dentata after stimulation of the perforant fibers as a mechanism of post-tetanic potentiation. Exp Neurol 1975;49:736–49. 173566 10.1016/0014-4886(75)90055-2

[btaf339-B57] Vidaurre-Gallart I , Fernaud-Espinosa I, Cosmin-Toader N et al A deep learning-based workflow for dendritic spine segmentation. Front Neuroanat 2022;16:817903. 35370569 10.3389/fnana.2022.817903 PMC8967951

[btaf339-B58] Vogel FW , Alipek S, Eppler J-B et al Utilizing 2D-region-based CNNs for automatic dendritic spine detection in 3D live cell imaging. Sci Rep 2023;13:20497. 37993550 10.1038/s41598-023-47070-3 PMC10665560

[btaf339-B59] Wagle S , Kraynyukova N, Hafner A-S et al Computational insights into mRNA and protein dynamics underlying synaptic plasticity rules. Mol Cell Neurosci 2023;125:103846. 36963534 10.1016/j.mcn.2023.103846 PMC10274545

[btaf339-B60] Wang Y , Wang C, Ranefall P et al SynQuant: an automatic tool to quantify synapses from microscopy images. Bioinformatics 2020;36:1599–606. 31596456 10.1093/bioinformatics/btz760 PMC8215930

[btaf339-B61] Yau K-W. Receptive fields, geometry and conduction block of sensory neurones in the central nervous system of the leech. J Physiol 1976;263:513–38. 1018277 10.1113/jphysiol.1976.sp011643 PMC1307715

[btaf339-B62] Yu Y , Adsit LM, Smith IT. Comprehensive software suite for functional analysis and synaptic input mapping of dendritic spines imaged in vivo. Neurophotonics 2024;11:024307 – 38628980 10.1117/1.NPh.11.2.024307 PMC11021036

[btaf339-B63] Yuste R , Bonhoeffer T. Morphological changes in dendritic spines associated with long-term synaptic plasticity. Annu Rev Neurosci 2001;24:1071–89. 11520928 10.1146/annurev.neuro.24.1.1071

[btaf339-B64] Zilberter Y. Dendritic release of glutamate suppresses synaptic inhibition of pyramidal neurons in rat neocortex. J Physiol 2000;528:489–96. 11060126 10.1111/j.1469-7793.2000.00489.x PMC2270153

