# Supplementary material for: SpyDen: simplifying molecular and structural analysis across spines and dendrites
Source: Bioinformatics. 2025 Jun 16;41(7):btaf339. doi: 10.1093/bioinformatics/btaf339 (PMC12233091; doi:10.1093/bioinformatics/btaf339)
Supplement: btaf339_Supplementary_Data [file btaf339_supplementary_data.zip › Supplemental_Material.pdf]

Supplemental material

SpyDen GUI

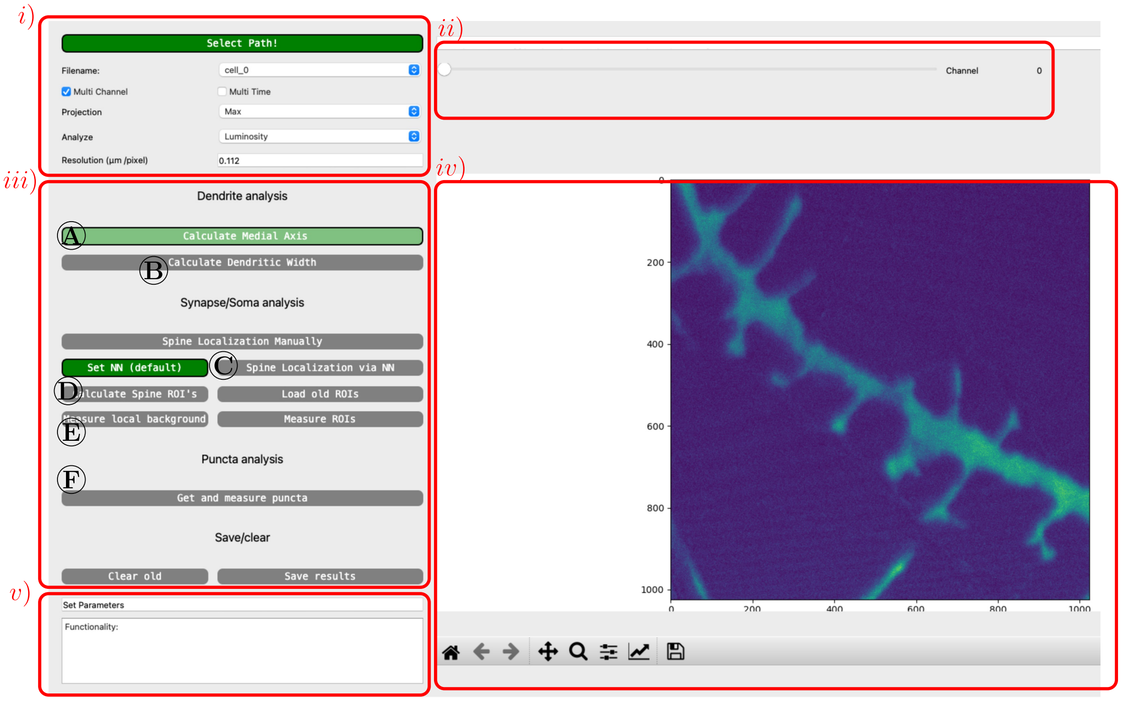

Figure S1: **Principle analysis window of SpyDen. We highlight the different components of this window in the red boxes and also show relate the analysis modes seen in Fig. 1 to the buttons that can be selected here.**

We emphasise that a full description of the GUI and data analysis pipeline of SpyDen can be seen in a set of video tutorials that we encourage the readers to watch.

The SpyDen GUI is the primary interaction point the user has with SpyDen. Coupled with a suite of video tutorials, SpyDen is easy to pick up and does not assume any programming knowledge to analyse experimental images. The primary analysis window is designed to provide the user with immediate feedback on what they can currently do, as each analysis step is only unlocked once all prerequisites are filled (green for active, grey for inactive). This step-by-step process leads to intuitive analysis, as logical and repeatable steps are performed for every data set.

The different components of the analysis are highlighted with red boxes in Fig. S1. Box i determines the meta-parameters of the data analysis, including the type of z-stack projection, image resolution and whether multiple channels are present. As the algorithms underlying the different analysis pipelines consist of tunable parameters that can affect the final result, we have introduced a set of sliders that appear for the relevant step in box ii. To select the different analysis modes, the user clicks the buttons in box iii. Box iv) provides feedback to the user by depicting the interactable image that can be clicked on, zoomed in on and analysed. Finally, box v is a text box that tells the user the current status of the analysis and what possible keyboard shortcuts they can use.

As the user proceeds through the analysis pipeline provided by SpyDen, the image in box iv changes to reflect the current status of the analysis. Examples of the different states of the image in box iv are depicted in Fig. 1B-G) with the buttons that lead to this behaviour marked in Fig. S1, iii). These include the dendritic analysis (A, B), synaptic selection and segmentation (C-E) and the puncta analysis (F). As

the image is built on an existing Python package (matplotlib), all the functionality of saving, panning and zooming are already implemented.

SpyDen data input

SpyDen takes *.tif*, *.lsm*, *.png* or *.jpeg* files as possible input. This choice of input formats was driven by the fact that the*.tif* format is some of the most widely used image formats in bio-sciences, while *.png* and *.jpeg* files are commonly used for general image files.

SpyDen then provides the option to alter the type of analysis that will be performed, e.g., the type of z-stack projection or whether to include temporal dynamics (to study such dynamics, the different time points need to be provided as separate images). SpyDen additionally requires the input of the experimental image's resolution (a necessary requirement for some of the subsequent analysis), which, if available, is obtained from the image metadata or provided manually.

Structure of the SpyDen output

**Dendrite_Channel_0.csv**	
**Column Name**	**Definition**
Dendrite: d	pair of x,y coordinates of the point on the medial axis path of dendritic ROI d
Width of ell.	width of the calculated ellipse at point x,y
Timestep t (Luminosity (mid.))[a]	Fluorescent intensity of the point on the medial axis at timestep t
Timestep t (Luminosity (ell.))[a]	Mean fluorescent intensity of all the pixels within the dendritic width (calculated using the ellipse approach) at timestep t

Table S1: Output file contains measurements for individual dendritic ROIs. [a] a separate column is added for each time step. A separate file is created for each channel as well.

Synapse_I_channel_i.csv	
**Column Name**	**Definition**
Id	Unique identification number assigned to each spine.
type	0 for stimulated spine, 1 for unstimulated spine and 2 for soma
location	pair of x,y coordinate of the centre of spine/soma on the image
bgloc	pair of x,y coordinate of the centre of corresponding background measurement on the image
area	area of the spine/soma polygon ROI in μm^2
distance	distance from the start of dendritic ROI along the medial axis path
Max. dist to Dendrite	Point on ROI polygon furthest from the Dendrite middle
Centre dist to dend	Distance of spine centre from dendrite middle
Min. dist to Dend	Point on ROI polygon closest from the Dendrite middle
Timestep t (mean)^a	mean of all pixel intensities inside the spine/soma ROI at timestep t
Timestep t (min)^a	minimum of all pixel intensities inside the spine/soma ROI at timestep t
Timestep t (max)^a	maximum of all pixel intensities inside the spine/soma ROI at timestep t
Timestep t (RawIntDen)^a	sum of all pixel intensities inside the spine/soma ROI at timestep t
Timestep t (IntDen)^a	mean intensity × area (in μm^2) of the spine/soma ROI at timestep t
Timestep t (bg mean)^b	mean of all pixel intensities inside the corresponding background ROI at timestep t
Timestep t (head_bbox)^c	Width and height of the minimum bounding box aligned perpendicularly to the dendrite encompassing the spine head ROI
Timestep t (neck length)^d	length of synaptic neck from the edge of the head ROI to the center of the dendrite or, if the width of the dendritic was generated, the width
Timestep t (neck width)^d	Average width of the spine neck
Timestep t (neck mean)^d	Average luminosity of the pixels inside the neck ROI
Timestep t (spine class)	classification of spine as mushroom or thin or stubby or outlier

Table S2: Output file structure generated for spine/soma ROIs when analysed using *luminosity* or *area* mode. ^{a}A separate column is added for each time step and one file per channel is created. ^{b}This is only generated in *luminosity* mode. ^{c}In *luminosity* mode, where there is only one ROI, this reduces to one single column. ^{d}These will only be populated if a suitable spine neck can be found.

Comparison of SpyDen capabilities with other open source and open access state of the art tools

dend_puncta.csv, soma_puncta.csv and neck_puncta.csv	
**Column Name**	**Definition**
Id	Unique identification number assigned to each punctum.
channel	Number of channel for which the punctum is located on
RoiId	Identification number of the dendritic ROI to which the punctum belongs
snapshot	Time point in a time series data to which the punctum belongs
location	Pair of x,y coordinate on the image for the punctum
radius	Radius of the circular punctum
max	Maximum fluorescent intensity of the punctum
min	Minimum fluorescent intensity of the punctum
mean	Mean fluorescent intensity of the punctum
std	Standard deviation of fluorescent intensities of the punctum.
median	Median fluorescent intensity of the punctum
distance	Distance from the start of dendritic ROI along the ROI

Table S3: output file containing measurements for individual punctum from puncta detection pipeline

Package Name	Codebase	ROI creation (Dendrites/ Spines/Puncta)	ROI Statistics (Dendrites/ Spines/Puncta)	Multi-channel anal.	Time-series anal.	GUI	Executable	Manual corrections	Output formats	2D/3D	Last upload (date)
SpyDen	Python	SA / A / A	✓/ ✓/ ✓	✓	✓	✓	✓	S, DI	.json, .csv, .roi, .npy	2D	Jul 2024
Neurobits [54]	MATLAB	M / M / A	✗/ ✗/ ✓	✓	✗	✗	✗	✗	.csv	2D	Jun 2018
StarSearch	MATLAB	M / M / A	✗/ ✗/ ✓	✓	✗	✗	✗	✗	.csv	2D/3D	Mar 2022
SynPAnal [9]	Java	M / M / A	✓/ ✓/ ✓	✓	✗	✓	✓	S,DI	.xlsx	2D	Mar 2015
Punctaspecks [51]	MATLAB	✗/ ✗/ A	✗/ ✗/ ✓	✓	✓	✓	✗	S	.csv	2D	May 2020
DeepD3 [15]	Python	A / A / ✗	✗/ ✗/ ✗	✗	✗	✓	✓	S	.tif, .hdf5, .roi	2D/3D	Mar 2024
SpineS [2]	MATLAB	A / A /✗	✗/✓/✗	✗	✓	✓	✗	DI	.mat, .csv	3D	Aug 2022
Spot Spine [21]	Java	✗/ SA / ✗	✗/✓/✗	✗	✗	✓	✓	S,DI	.swc, .csv	3D/2D	Jun 2024
AUTOTUNE [62]	MATLAB	SA / SA / ✗	✓/✓/✗	✗	✓	✓	✗	S	.mat	2D	Jun 2024
SpineTool [14]	Python	✗ / A /✗	✗/✓/✗	✗	✗	✗	✗	DI	.off, .csv	3D	Jul 2023
RESPAN [19]	Python	A / A / ✗	✗/✓/✗	✗	✓	✓	✓	✗	.csv	3D/2D	Aug 2024
SENPAI [5]	MATLAB	A / A /✗	✓/✓/✗	✗	✗	✓	✗	✗	.mat	3D	Mar 2024
DeepSpineTool [57]	Python	A / A /✗	✓/✓/✗	✗	✓	✓	✓	✗	.tif	3D	Mar 2022
SpineJ [34]	Java	A / A / ✗	✗/✓/✗	✗	✓	✓	✓	DI	.roi	2D	Mar 2020
SynQuant [60]	Java	A / A / ✗	✓/✓/✗	✓	✗	✓	✓	✗	.roi	2D/3D	Jul 2020
3dSpAn [10]	C++	A/A/✗	✗/✓/✗	✗	✗	✓	✓	S	.csv,.tiff, .img	3D	Jul 2022
SynActJ [49]	Java	✗/ A /✗	✗/✓/✗	✗	✓	✓	✓	✗	.csv	2D	Dec 2021
SynBot [47]	ImageJ Macro	✗/ A / ✗	✗/ ✓/ ✗	✓	✗	✓	✓	✗	.csv, .tif	2D	Dec 2024

Table S4: A non-exhaustive list of tools for dendrite, spine and puncta analysis literature from 2015 to 2024. Abbreviations; SA: Semi-Automated (requiring minimal manual input); A: Automated (without requiring any manual input), M:Manual; S: Sliders, DI: Direct Intervention; ✗: Not supported, ✓: Supported

Effect of the algorithm parameters on the dendritic segmentation

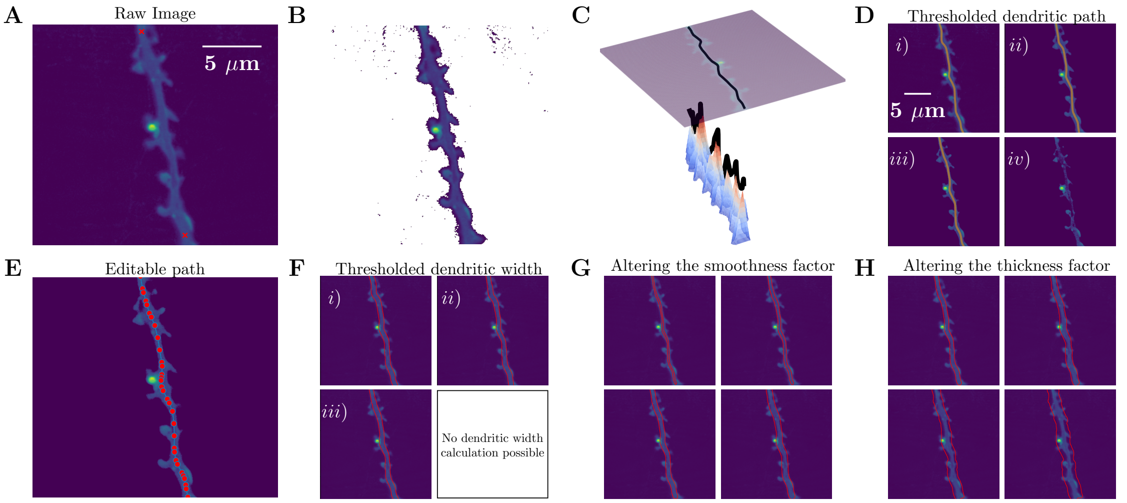

Figure S2: **Applying the median threshold to the raw image allows us to generate better medial axis paths for the dendrites and subsequently better approximations of the dendritic width.** **A)** Example input image that is provided to the code with start and end points of the dendritic segmented marked in red by the user. **B)** Given this image, SpyDen generates a filtered version of the image, where all noise is removed from the image and only key features are retained. Here, we use the default threshold value. **C)** Given this filtered image SpyDen generates a weighted matrix (see 3D plot) where it finds the shortest path that traverses the highest points. These highest points represent the centre of the dendrite. **D)** By using different threshold values, more or less features are filtered out. This leads to slightly different optimal dendritic paths (marked with the orange line). Each of the 4 sub-images (*i* to *iv*) depicts a slightly higher user-set threshold. We note that case iv) filtered too much of the dendrite out and so no optimal path was found. **E)** Once a suitable path is calculated, the user can make fine corrections to the code by moving editable nodes along the suggested path (highlighted by the red circles). **F)** Using the selected threshold and dendritic path a dendritic width can be calculated (red lines on either side of the dendrite). We note that it was not possible to find the dendritic path and subsequently the dendritic width for case iv) as the threshold eliminated significant parts of the dendrite. Thus, there is no viable path between the start and end points. **G)-H)** A set of tunable parameters allow for user interaction with the dendritic width calculation. These are the smoothness factor in (G), which determines the amount of pixels that are used to calculate the length of the outward pointing normal from the medial axis and the width multiplication factor in (H) which multiplies the outward pointing normals by a set value. Altering these values can have a substantial effect on the calculated width and allow for a significant amount of flexibility for the user. The threshholded dendritic with from *F*iii) was used.

Neural network approach to spine identification

To train the neural network, three separate datasets were employed. Examples of these images can be seen in Figure S4. More concretely the details of the images can be seen as follows:

Dataset	Type of cell	Resolution	Reference	Use in network
Helm	Hippocampal cultured neurons	0.02021 μm	[27]	Training + Testing
Chater	Organotypic hippocampal slice culture neurons	0.66 μm	[6]	Training + Testing
Deep3D	Organotypic hippocampal slice culture neurons	0.094 μm - 0.1245 μm	[15]	Training
Cultured	Hippocampal cultured neurons	0.29 μm	N/A	Testing

Table S5: Experimental details of the network images used in the spine detection ANN

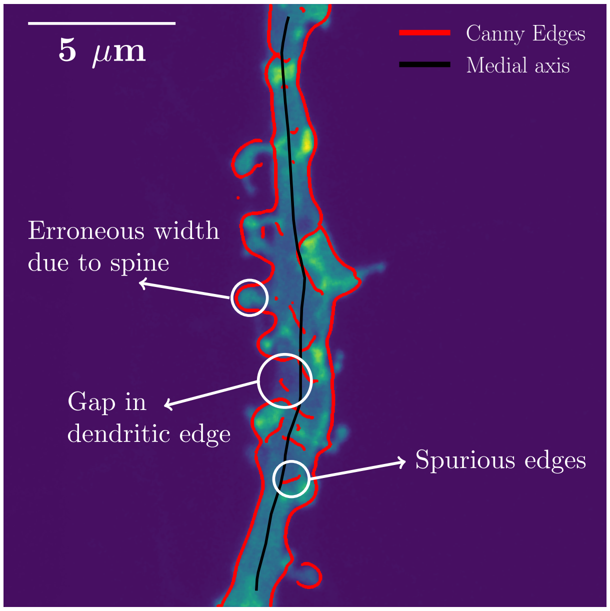

Figure S3: Applying the canny-edge detection algorithm to a filtered version of the experimental images provides an acceptable set of dendritic edges. However, as illustrated by the white circles, several problems preclude using the edges directly. Instead, we apply the ellipse approach seen in Fig. 2A-E) to enhance the dendritic segmentation.

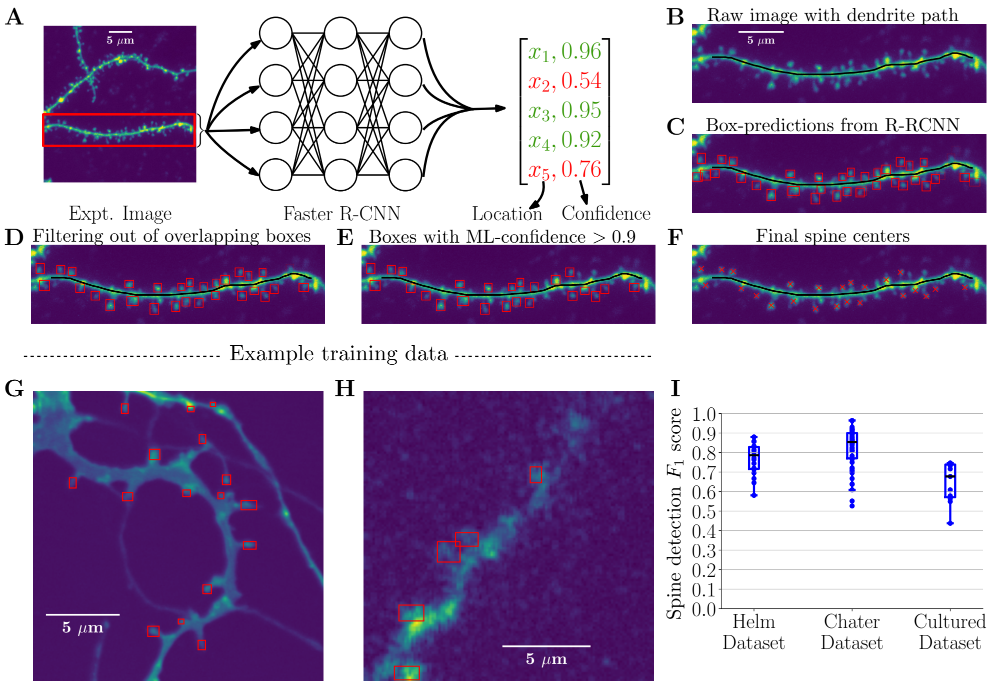

Figure S4: **Depiction of the neural network approach with example images used in the spine detection in SpyDen.** **A)** Schematic depicting a simplified view of the neural network that is described in (*C*-*F*). Initially, a bounding box is drawn around the dendritic stretch of interest (also seen in *C*). Then, this information is fed to a pre-trained neural network which outputs a list of bounding boxes within that dendritic and the associated confidence of the neural network. The user can then choose to filter the output of the network based on the confidence. Here, we have set the confidence threshold at 0.9, so the suggested points x_2 and x_5 are excluded from further analysis. **B)** Example input that is fed into the neural network. Depicted in black is the medial axis path calculated in the tool as part of the previous step of the analysis. **C)** Using this image, the pre-trained neural network (based on a Faster R-CNN architecture [22], but modified for our purposes), we are then provided a set of bounding boxes and confidence scores for each proposed identified spine. The opacity of each box is defined by the confidence of the algorithm. **D)** When the algorithm suggests multiple overlapping bounding for the same spine, only the bounding box with the highest confidence is selected. **E)** As part of the neural network approach, the user can filter out bounding boxes below a certain confidence score. Here, we have taken the boxes from (c) and filtered out those that have a confidence level less than 0.9. **F)** Finally, using the bounding boxes calculated in (d), we can generate the suggested spine centres (marked with a red cross). These points are then used to generate the bounding ROIs for the subsequent analysis steps. **G, H)** Example images (with manually generated bounding boxes) taken from the two additional datasets used to train the SpyDen spine detection network. These datasets are the Helm dataset [27] and Deep3D [15] datasets. **I)** Evaluation of the spine centre detection of the SpyDen neural network using the F_1 metric. We note that the automatic procedure to generate spine centres (and which can be augmented with manual selection/deletion) leads to reasonable results across the three different test datasets.

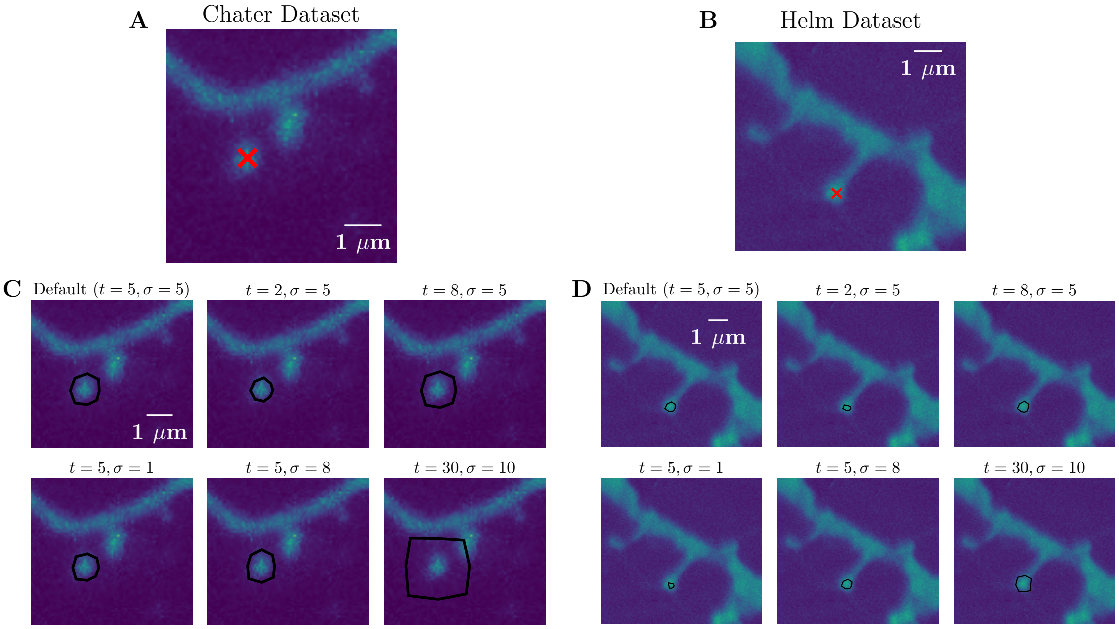

Figure S5: **Tuning the parameter settings allows for the automatic ROI generation to adapt to a variety of experimental images.** **A)-B)** Example experimental spines marked with a red cross from the [6] and [27] datasets, respectively. **C-D)** Given these spines and the marked locations, the code can generate a set of different ROI given different values of the tolerance t, and σ of the canny edge detection filter. We note that certain parameter combinations work better for certain datasets (e.g., the default setting generates a satisfactory ROI for the Chater spine, while the $t = 30, \sigma = 10$ generates the correct ROI for the Helm spine. All these of ROIs are automatically generated and have not been edited using the editable nodes.

The effect of noise

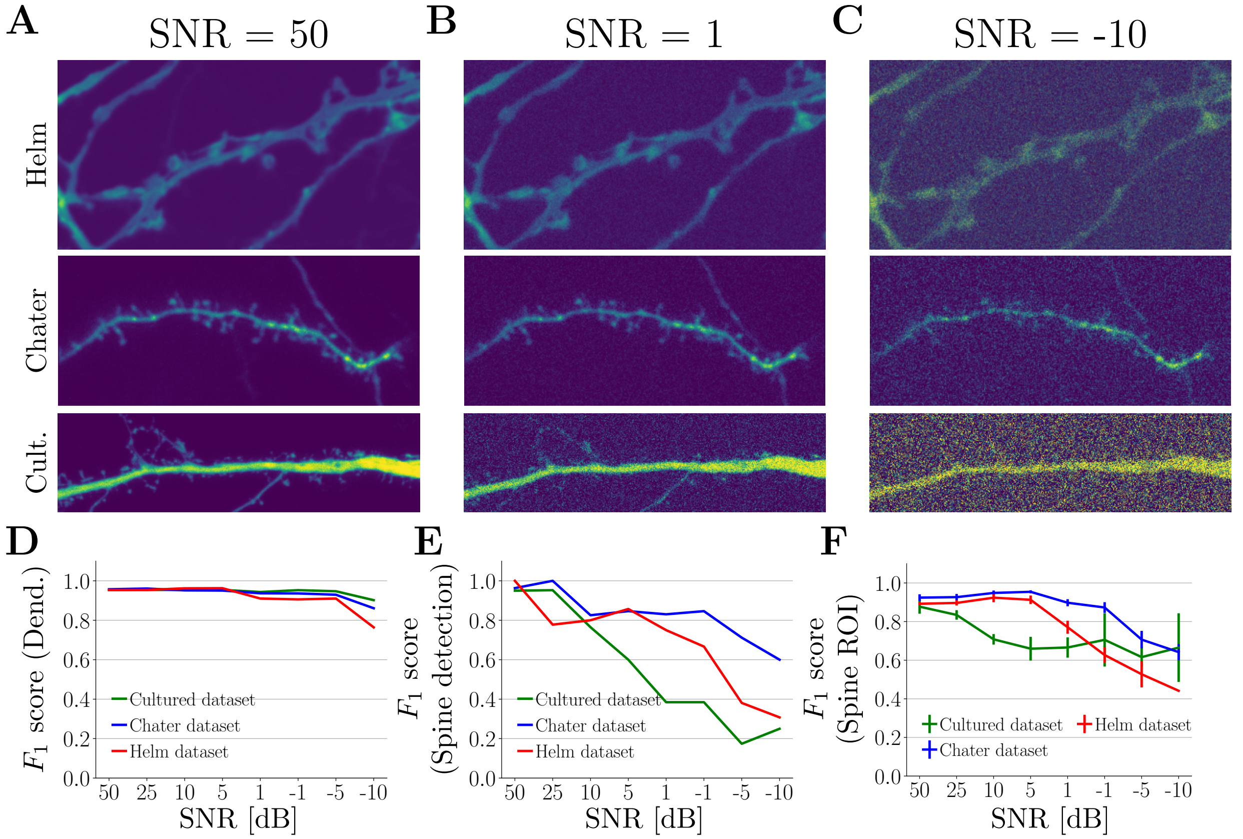

Figure S6: **While simulating noisy experimental conditions with SNR values ranging from 50 to -10db, the automatic SpyDen algorithms are able to achieve robust results. A-C)** Taking a representative image from each of the dataset and adding Gaussian noise allows us to generate different noisy experimental conditions. **D-F)** Using the noisy images, we can use the SpyDen results from the raw image as a baseline and compute the F1 scores of (D) the dendritic segmentation, (E) the detection of spine heads and (F) the spine ROI segmentation for the detected spines. In all cases, the algorithm parameters were set once and applied to all SNR conditions. Error bars in E represent the SEM across the different spines.

Individual datasets

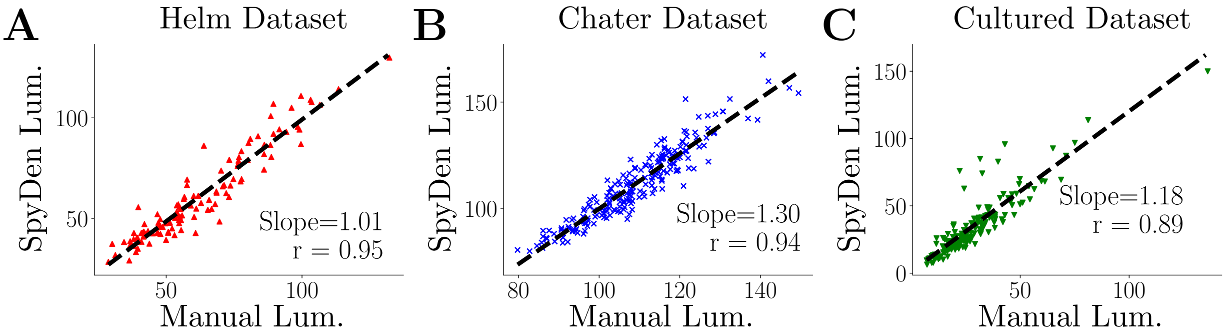

Figure S7: **SpyDen agrees across a variety of different experimental conditions. A-C)** Comparison of the luminosity calculated using the manual ROIs against the luminosity of the SpyDen ROIs for Helm, Chater and Cultured dataset, respectively. In each case, a linear fit is overlaid showing good agreement between the two evaluations.

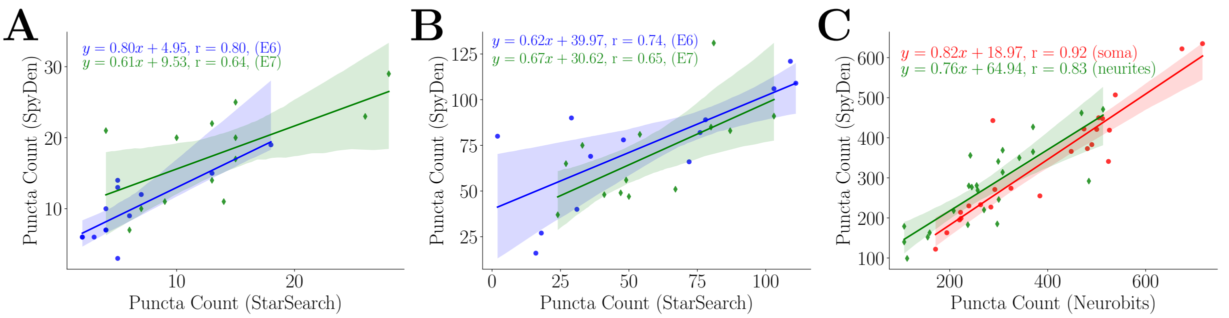

Figure S8: **SpyDen puncta detection is in good agreement with other specific-tools across multiple experimental conditions. A-C)** Comparison of the puncta count in smFISH from ([8]) for different isoforms of cdc42 using StarSearch and SpyDen in Neurites (A) and Soma (B) ROIs . C) Comparison of puncta count in FISH images from ([16]) using SpyDen against Neurobits. In each case, a linear fit is overlaid (with 95% confidence Intervals) showing good agreement between the two evaluations.

Algorithms

Algorithm 1 Finding of the dendritic medial axis path

Require: : Image of dendrite and spines, I, threshold t, start point p_s and end point p_e

Perform a threshold of I with value t to obtain a binarised image E of dendrite and spines.
for each pixel e_{ij} with pixel coordinates ij in E **do**
  if $e_{ij} == 1$ **then**
    create a node n_{ij} in the Graph G. ij are the preserved coordinates of the nodes.
  end if
end for
Cost map for shortest path
for every node n_{ij} in G **do**
  $n_{i'j'}$ is nearest neighbour with $i'j' \in \{(i+1)j, i(j+1), (i+1)(k+1), ...\}$(8 combinations)
  if $n_{i'j'}$ exists **then**
    create edge $e_{iji'j'}$ between nodes n_{ij} and $n_{i'j'}$ with value $|(i-i', j-j')|$ (1 or $\sqrt{2}$) in Graph G
  end if
end for
Cost Map for following boundaries
Make a copy G' of G and set incrementer for every node $i_{i'j'}$
Make a copy G'' of G'
while nodes $n''_{i''j''}$ exist in G'' **do**
  for all nodes $n''_{i''j''}$ in G'' **do**
    if number of edges for $n''_{i''j''} < 8$ **then** remove node $n''_{i''j''}$ and corresponding edges
    end if
    if $n_{i''j''}$ in G'' **then**
      set $i_{i'j'} = i_{i'j'} + 1$
    end if
  end for
end while
invert assigned values $i_{i'j'}$ in G'
Cost Graph $C = \lambda G + \eta G'$ with $\lambda + \eta = 1$
Perform Dijkstra algorithm on C from node p_s to p_e to obtain medial axis path which penalises walking on boundaries.

Algorithm 2 Finding of the dendritic width

Require: : Image, I, Set of spatially ordered dendritic points on the medial axis path , $\vec{d}$, Canny edge variance, σ

Perform a canny image detection on I and generate a Boolean matrix C of the contour of the dendrite
Create a place holder array W for the width data of size length($\vec{d}$) x 4
incrementer $i = 0$
for each point d_i in $\vec{d}$ **do**
    Create a boolean matrix E of an ellipse ϵ with the centre d_i
    Minor axis in the direction of the next neighbour d_{i+1} with a fixed radius of $r_{min} = 2\,\text{Pixel}$
    For the major axis radius we set an incrementer $r_{maj} = 1\,\text{Pixel}$ and a counter $c = 0$
    while sum(Intersection(C, E) < 1 or $c < 30$ **do**
        Create a new E with $r_{maj} = 1.2 \cdot r_{maj}$
        $c = c + 1$
    end while
    The angle of the ellipse can be calculated with $a = arcsin((d_{i+1} - d_i)_x r_m in/(r_{min}))$
    $W_i = (d_i,\, r_{min},\, r_{maj},\, a)$
    i = i + 1
end for
A Maximum increase condition of the width within two neighbours of the medial axis path is applied
From W the boolean mask of the segmented dendrite can be obtained

Algorithm 3 Finding the region of interest for a spine

Require: : Image, I, Spine point, s_0, Canny edge variance, σ, Luminosity parameters, a, b, background luminosity l_{bg}, n, number of rule breaks

Perform a canny image detection on I and generate a Boolean matrix E of edges
if $E[s_0]$ is not an edge **then**
    Set $c_k = 0$ for all directions, $k \in d$
    Set incrementer $i = 1$
    Obtain luminosity, l_0, for point s_0
    while $c_k \leq n$ for at least one k **do**
        Set $d' = d$ where $c_d < n$
        for $k \in d'$ **do**
            $s_{k,i} \leftarrow s_0 + in_k$
            Apply the ROI rules to $s_{k,i}$ adding to c_k as necessary.
        end for
        $i \leftarrow i + 1$
    end while
    Gather points s_k as the full octagonal ROI, R_0
    Generate points s_n, s_e, s_w, s_s as the offsets of s_0 (one pixel in each cardinal direction)
    Restart the algorithm for the new points and obtain ROIs R_n, R_e, R_w, R_s.
    Return Average$(R_0, R_n, R_e, R_w, R_s)$
end if

Algorithm 4 Spine Classification Algorithm

Input S: array of segmented spines in image I
Output: Classification of each $s\_i \in S$ as stubby, mushroom, thin or outlier
for each $s\_i \in S$ **do** do
  if $S_i.neck\_width == 0$ **then**
    s_i = stubby
  else if $\frac{S_i.neck\_width}{S_i.head\_width} < 0.5$ **then**
    s_i = mushroom
  else if $0.5 < \frac{S_i.neck\_width}{S_i.head\_width} < 1.1$ **then**
    s_i = thin
  else
    s_i = outlier
  end if
end for

ROI generation rules

The rules used for the generation of the synapse ROIs (and described briefly in the main text and the algorithm above) are presented here in a detailed manner:

1. **Boundary Rule**
   If $s_{d,i}$ exceeds the boundary of the image, the progression in this direction is immediately halted, i.e., c_d is set to be n.

2. **Contour Rule**
   The image is treated with a Canny edge detection algorithm that takes a pre-determined sigma parameter that defines the Gaussian kernel and retrieves edges in the subject matter. If the $s_{d,i}$ encounters one such edge, this adds a certain number of strikes to c_d depending how far we are from x_o

3. **Luminosity Fall-off Rule**
   If the luminosity, $l_{d,i}$ becomes luminosity, l_0/a or the luminosity falls below b times the background luminosity l_{bg}, the algorithm adds one to c_d, i.e., you continue while

$$l_{i,j} \geq \frac{l_0}{a} \tag{1}$$

$$l_{i,j} \geq b l_{bg} \tag{2}$$

4. **Dendrite Rule**
   We assume that the spine, on average, is symmetrical and that the user will supply the centre of the spine for (x_0, y_0). Therefore, if $v_{i,j}$ is closer to the dendrite centre than the initial point, c_d is also incremented.

5. **Symmetry Rule**
   As a direct consequence of the previous assumption, we also introduce the symmetry rule: the ray directly opposite cannot be more than twice as long before triggering a strike. Assuming that $s_{-d,i}$ is a ray that has stopped progressing (i.e., $c_{-d} \geq n$), we can mathematically describe the moment that one is added to c_d

$$s_{d,i} > s_{-d,i} \tag{3}$$

6. **Luminosity Increase Rule**
   Another assumption of the algorithm is that point s_0 is among the brightest pixels within the spine. Thus, any consistent increase in luminosity is indicative that we have entered the dendrite or another spine. Therefore, persistent luminosity strengthening also leads c_d being increased by one.

7. **Overlap Rule**
   We note that ROIs of different spines should not overlap, as then some quantities will be counted at least twice. Therefore, given another spine location s_0', the distance between $s_{d,i}$ and s_0 must be less than to s_0'.

This simple set of rules leads to a small set of parameters for the user to use, meaning that robustness and reproducibility are maintained across a wide array of experimental conditions. These parameters are as follows:

1. The threshold n that defines when the ray progression ends
2. The value of variance, σ of the Gaussian filter in the canny edge detection
3. The values a and b of the luminosity thresholds.

The initial values that have worked well for the examples in this article are set to $n = 3$, $\sigma = 1.5$, $a = 3$ and $b = 4$. However, the option to change these values and turn on and off certain features in the ROI generation allows for a broader use case where certain assumptions or rules may not be valid.
