# Supplementary material for: SpyDen: simplifying molecular and structural analysis across spines and dendrites
Source: Bioinformatics. 2025 Jun 16;41(7):btaf339. doi: 10.1093/bioinformatics/btaf339 (PMC12233091; doi:10.1093/bioinformatics/btaf339)
Supplement: btaf339_Supplementary_Data [file btaf339_supplementary_data.zip › User_manual.pdf]

# User Manual for SpyDen tool

Version 1.3.0-beta

Apr 2025

This is a user manual that provides a brief introduction on how to operate the SpyDen tool. For more details and in-depth discussion of the underlying algorithms, we refer you to our paper SpyDen: Automating molecular and structural analysis across spines and dendrites.

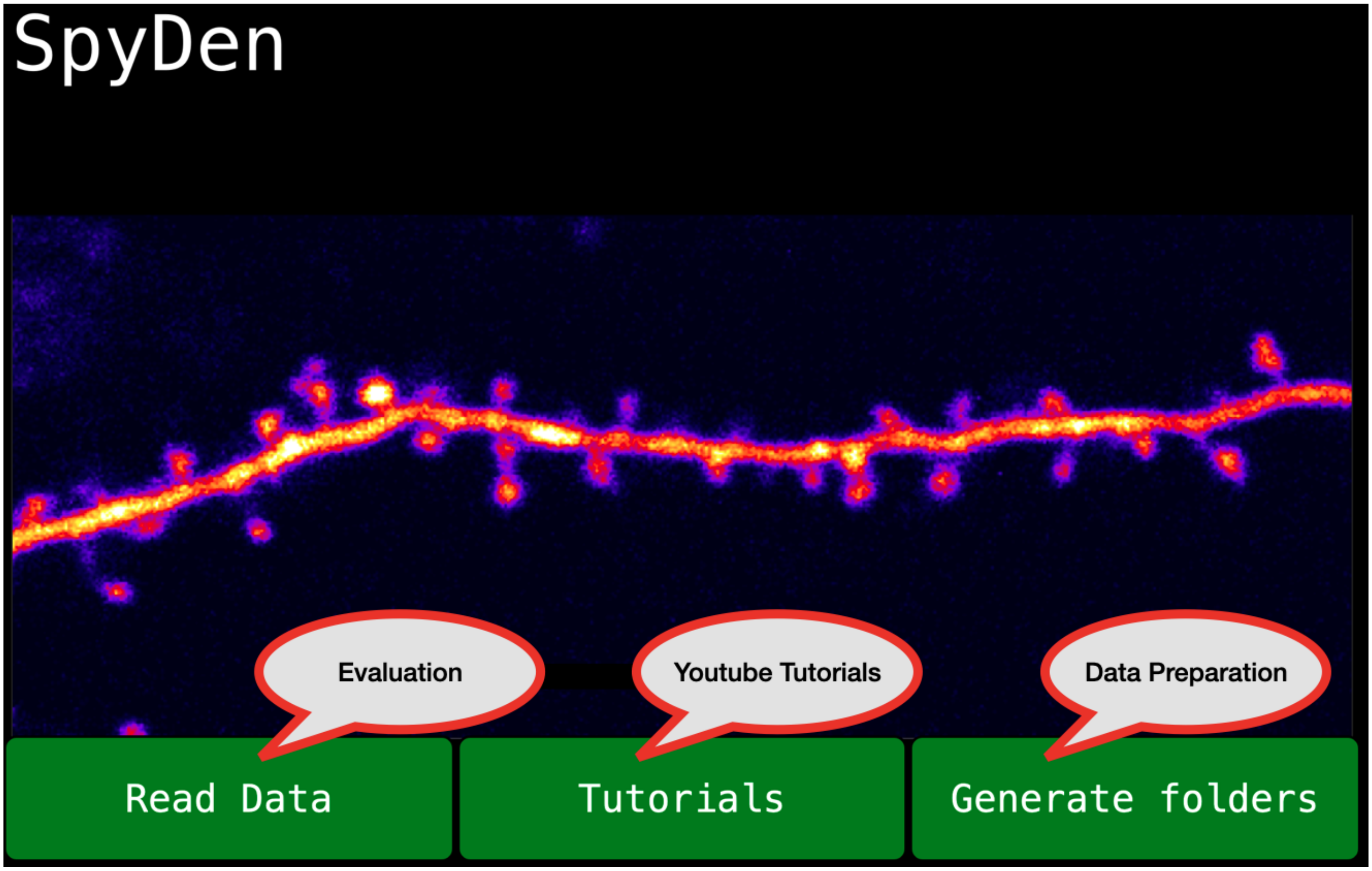


## 1 Data Preparation

SpyDen takes a variety of images as input including:

- *.png*
- *.lsm*
- *.tiff*
- *.jpeg*

In the chase that you wish to provide multiple channels, please provide an image where this is the first dimension. SpyDen will then perform a z-projection (if there is one), to generate an image with the following dimensions: [**c, x, y**]. If the user wants to study dynamic data which includes different time steps, then they should provide each timepoint as a separate image. SpyDen will read these and attempt to arrange them by ordering their file names in ascending order. This leads to the extra dimension **t**. To summarize:

- **c**: Channel type (e.g., morphological channel, Homer1 protein distribution, etc.).
- **x**: Horizontal axis of the image (pixels along the x-direction)
- **y**: Vertical axis of the image (pixels along the y-direction)
- **t**: Time axis (frame index for time-resolved data)

### 1.1 Folder Structure

SpyDen provides a way to generate the correct folder structure as input, while protecting your raw data from alteration/deletion. For this purpose you use the ”Generate folders” button, which will allow you to choose your data (where each experiment should be in a separate folder) and a final location to save. However, if you wish to generate the data structure for SpyDen yourself and to ensure a uniform and automated workflow, the input data must be organized as follows:

1. Create a main folder containing all cell image data.
2. For each cell, create a dedicated subfolder, for example, **cell_1**, **cell_2**, **cell_3**, and so on.
3. Store the input images files within these subfolders.

   **Tip**: If you want an introduction to how SpyDen works or a visual demonstration of the analysis process, feel free to watch our YouTube Video.

## 2 Evaluation

To begin, click the **Read Data** button. This opens the main window, which remains your central interface throughout the analysis process. Importantly, SpyDen will guide you through the analysis process by only making buttons/options active that are currently executable. In other words, when an action is possible the corresponding button will become green, otherwise it will be grey. Additionally, sliders that are only relevant for the current analysis mode (e.g. timestep slider) will only appear when they are relevant.

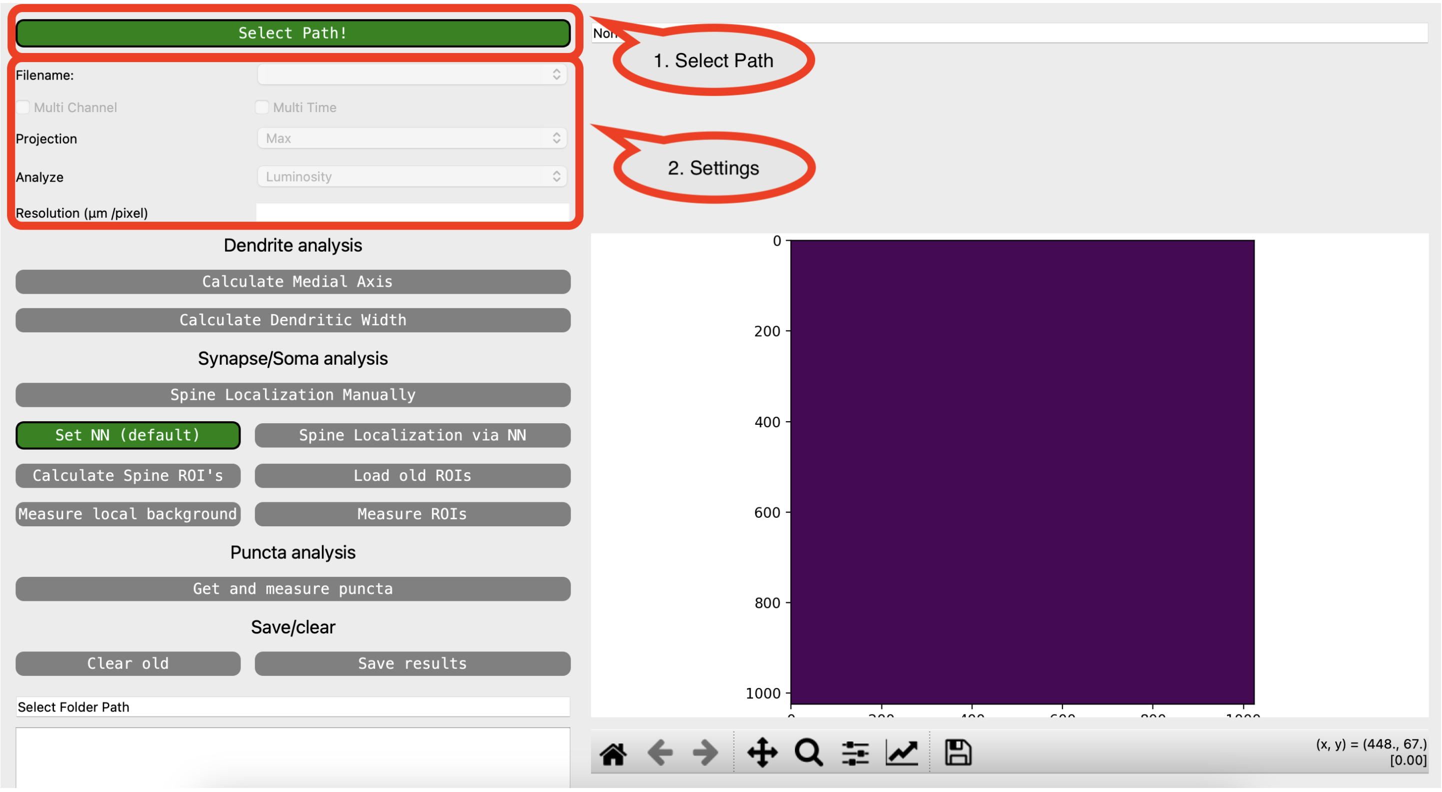

## 2.1 Initial Settings

1. **Path Selection**
   In the top-left area, select the directory that contains your cell subfolders. If you are using the software for the first time, we recommend starting with the example dataset found in the **TestImages** folder.

2. **Filename**
   Specify the name of the cell (i.e., the subfolder), such as `cell_1`.

3. **Multi-Channel / Multi-Time**
   Enable these options if your dataset includes multiple fluorescence channels or multiple time frames.

4. **Projection**
   Choose the desired projection type (maximum projection, minimum projection, sum, mean, etc.). This is crucial for 3D stacks.

5. **Analysis**
   If you want to study spine head ROIs, then you can choose two analysis modes - "Area" or "Luminosity". These behave in fundamentally different ways:

   - Area: Will generate a ROI around the spine head for each timepoint.
   - Luminosity: will generate a ROI that surrounds the spine head for all timepoints.

6. **Resolution**
   Indicate the microscope resolution used to acquire the images (e.g., STED, confocal microscopy). This information is relevant for proper scaling and quantitative analyses later on. Where possible SpyDen will try to read this information from the image metadata.

After entering these settings (which are saved in the Settings file for each individual experiment), you can begin the analysis.

> **Tip**: There is a global settings file, which will save SpyDen settings between sessions and apply these to a new experiment if you have not analysed it yet. However, if you want to have a consistent set of settings across a set of experiments/cells, you can copy the *setttings.txt* file into all the folders you want to analyse and the settings will be automatically imported.

# 3 Dendrite Analysis

Once the image stack is loaded, SpyDen provides two main features for dendritic analysis:

1. **Calculation of the Medial Axis Path**

2. **Dendrite Segmentation** including width determination

The dendritic segmentation can only be performed once the medial axis path has been completed.

## 3.1 Calculating the Medial Axis Path

The **medial axis** of an object roughly corresponds to its "central skeleton." For dendrites, this representation offers a robust way to determine their overall course.

1. **Set Threshold**
   Use the slider in the top-right corner to select an appropriate threshold value that separates the foreground (dendrite) from the background, providing a coarse segmentation. SpyDen will attempt to find this threshold itself if possible, but it may need to be adjusted.

2. **Place Start and End Points**
   With a **left-click**, first select a start point and then an end point for the dendritic section you wish to examine. As soon as the second point is set, SpyDen automatically computes the medial axis path.

   > **Tip**: If you have generated a medial axis path already using a different software, you can use the **Load Dendrite Path** button as long as it is in .npy or .roi format.

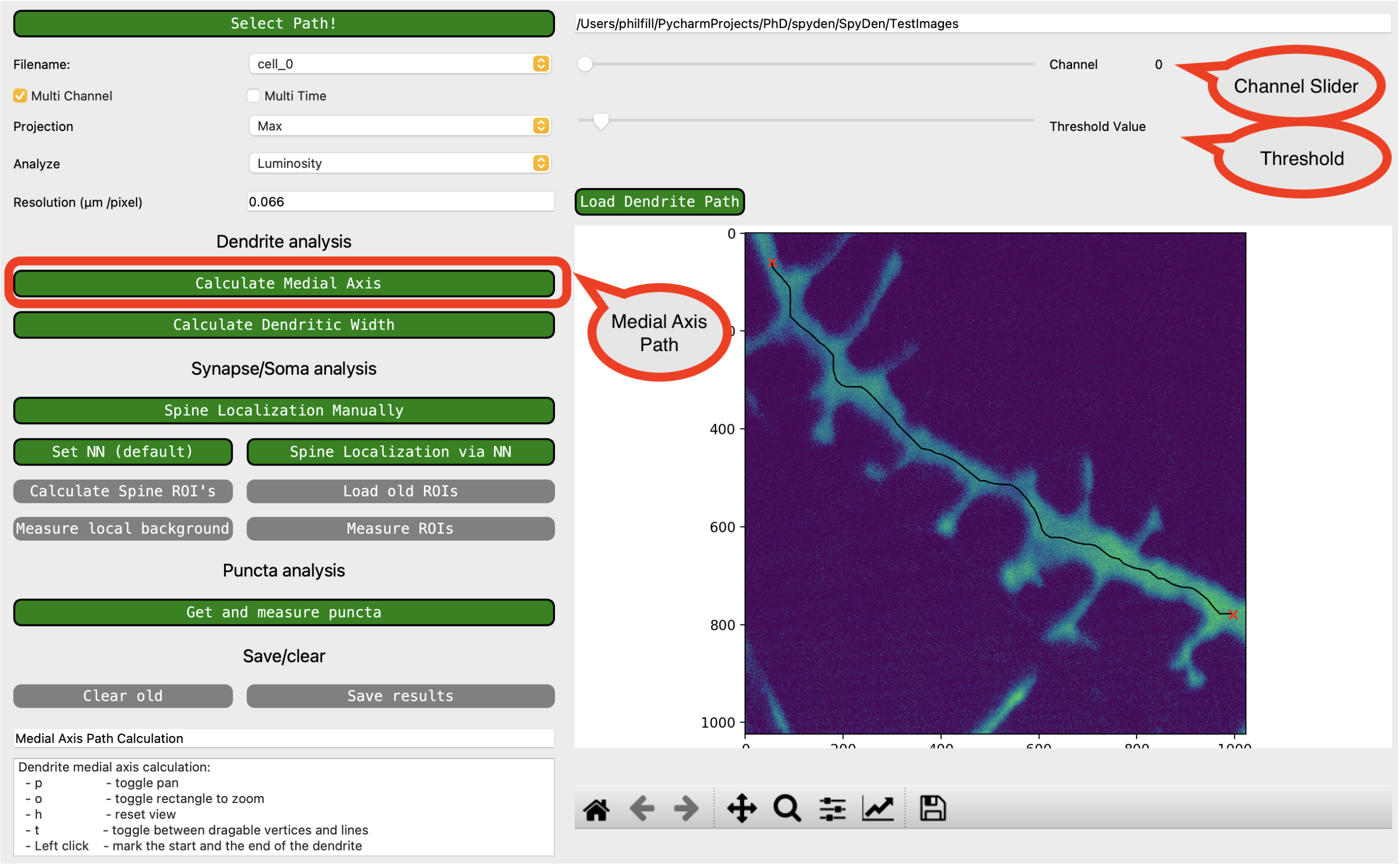


1. **Analyzing Multiple Dendrites**
   If your image contains multiple dendrites, simply repeat the process (select start and end points). Each dendritic section is displayed and stored separately.

### Useful Shortcuts

- **Left-click**: Defines the start and end points of the dendrite segment to be analyzed.
- **p**: Toggles between panning mode and interactive mouse mode.
- **o**: Activates a rectangular zoom tool for focusing on a specific image region.
- **h**: Resets the view to the default position and magnification level.
- **d**: Deletes a marked point (hover over the point and press **d**).
- **Backspace**: Clears all annotations, including points and paths, from the current image.
- **t**: Opens the medial axis path adjustment mode, allowing for manual refinement of control points.
  - **i**: Inserts a new control point (hover over a line segment and press **i**).
  - **d**: Deletes a selected control point (hover over the control point and press **d**).
  - **Left-click**: Enables dragging of control points (click and hold on a control point to move it).

## 3.2 Manually editing the calculated path

If the automatically calculated medial axis path does not meet your requirements, you can manually refine it using the adjustment mode. In this mode, the medial axis is represented as a piecewise linear polygon defined by control points. These control points can be interactively modified via drag-and-drop to achieve the desired path geometry.

**Useful Shortcuts**

- **t**: Opens the medial axis path adjustment mode, allowing for manual refinement of control points.
- **i**: Inserts a new control point (hover over a line segment and press **i**).
- **d**: Deletes a selected control point (hover over the control point and press **d**).
- **Left-click**: Enables dragging of control points (click and hold on a control point to move it).

## 3.3 Dendritic Width

Once the medial axis path has been determined, you can measure dendritic width. SpyDen uses a series of ellipses placed along the axis:

1. **Ellipse at Each Axis Point**
   At each point along the medial axis, an ellipse is placed such that its **minor axis** aligns with the local direction of the dendrite, while its **major axis** extends outward radially.

2. **Boundary Detection**
   The major axis is increased until it reaches the dendritic boundary (defined by the threshold segmentation). The union of all these ellipses describes the complete dendritic segmentation.

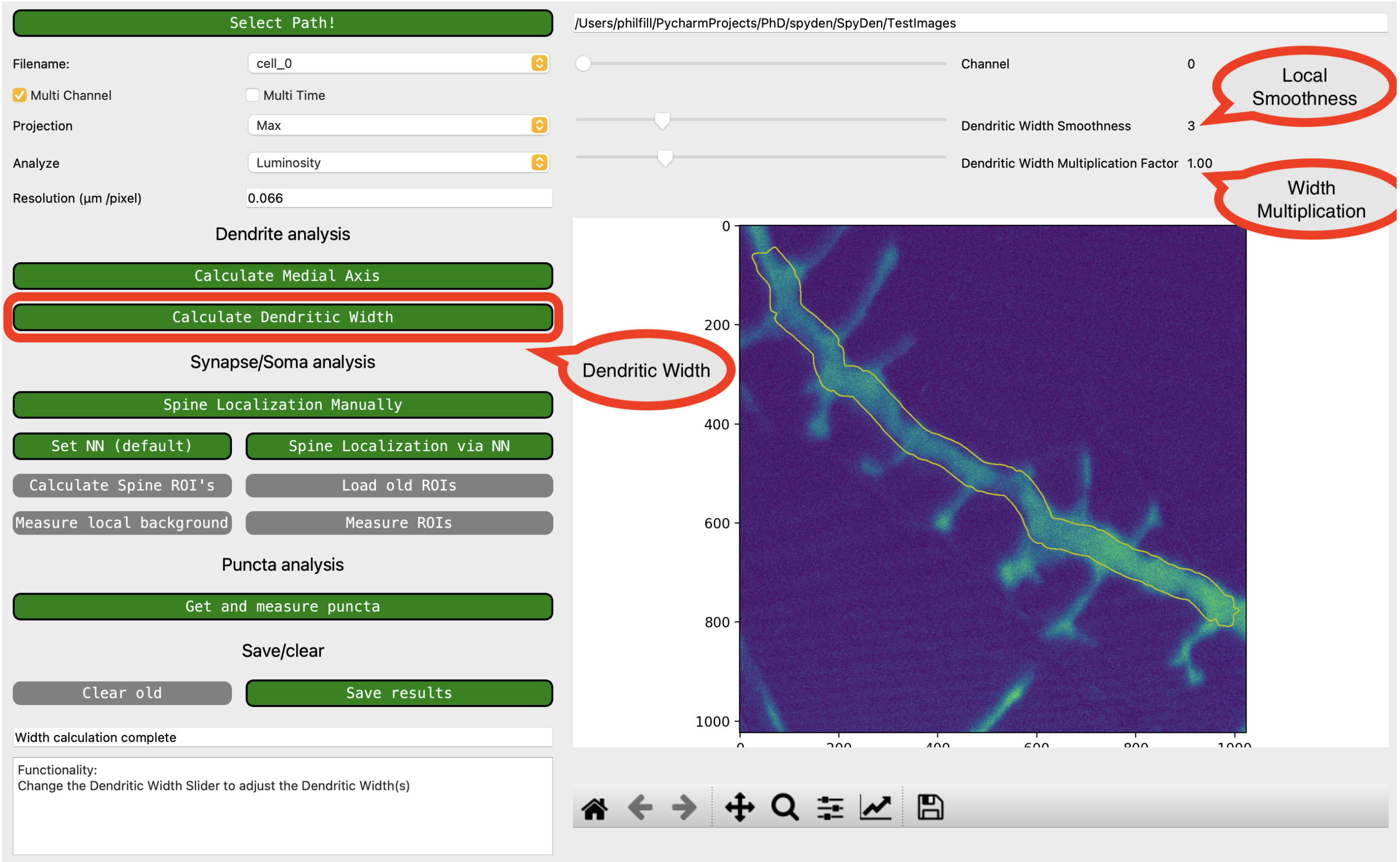


- **Dendritic Width Smoothness**
  This parameter limits how much the maximum width (radius) can change between neighboring points along the medial axis, preventing abrupt and unrealistic width changes.
- **Dendritic Width Multiplication Factor**
  Scales the measured dendritic width globally for all points. This allows you to correct for complex datasets or make fine adjustments to the overall segmentation.

### 3.4 Dendritic Output

After the analysis of a dendrite, the results can be saved. Several output files are generated for this purpose. In the folder corresponding to your exemplary cell, a subfolder named **Dendrite** will be created.
Inside this folder, you will find a dendrite mask named **Mask_dend$x$.png**, where $x \in \mathbb{N}$ denotes the $x$th dendrite. Furthermore, files named **Dendrite_Channel_$i$.csv** are provided, where $i \in \mathbb{N}$ refers to the $i$th image channel. Each CSV file contains the individual pixel values $f(x, y)$, where $x$ and $y$ represent the horizontal and vertical coordinates within the image, respectively.
In addition, for each dendrite a file named **Dendrite$i$.npy** is generated, where $i \in \mathbb{N}$ denotes the $i$th dendrite. This file contains the medial axis path represented as a sequence of pixel coordinates $(x, y)$. For each point along the medial axis, an associated ellipse is defined, characterized by its major and minor axis lengths as well as the rotation angle relative to the horizontal (x-) axis. These ellipses provide a local geometric approximation of the dendrite's cross-sectional shape at each point along its path.
Additionally, a file named **Dendrites.json** is generated, which contains quantitative and positional information for each analyzed dendrite. Each entry in this file includes the start and end coordinates of the dendrite, the total dendritic path length, and several statistical measurements computed across all channels.

For each image channel, the following parameters are recorded:

- **Mean**: The average pixel intensity along the dendrite.
- **Area**: The area in pixels of the dendritic mask.
- **Min** and **Max**: The minimum and maximum pixel intensities, respectively. These values represent the extrema found in the projection of the image along the dendritic axis.
- **Raw Integrated Density**: The sum of raw pixel values within the dendritic region.
- **Integrated Density**: The background-corrected integrated pixel intensity.

This structured data format allows for a comprehensive evaluation of dendritic signal characteristics across multiple fluorescence channels and can be used for further quantitative analysis or automated processing workflows.

**Result**: After this step, you have a fully automated segmentation and width measurement of the dendrite. You can now proceed with spine analysis or use the dendrite data for further evaluations.

## 4 Spine Analysis

This section explains how to detect and segment spines. Before proceeding, please ensure that you have downloaded our pre-trained **Mask R-CNN** network and set it up correctly, if you wish to use automatic detection. This can be done using the **Download NN** button.

> **Tip**: If you want to provide your own neural network you can, but please reach out to us (you can contact us through the SpyDen software in the Tutorials button) and we can help you get that working.

### 4.1 Spine Localization

SpyDen provides two methods for locating spines:

1. **Manual Localization**
   Users mark each spine head by clicking on it.
2. **Automatic Localization**
   The pre-trained Mask R-CNN detects spines based on features learned from approximately 20,000 annotated spines.

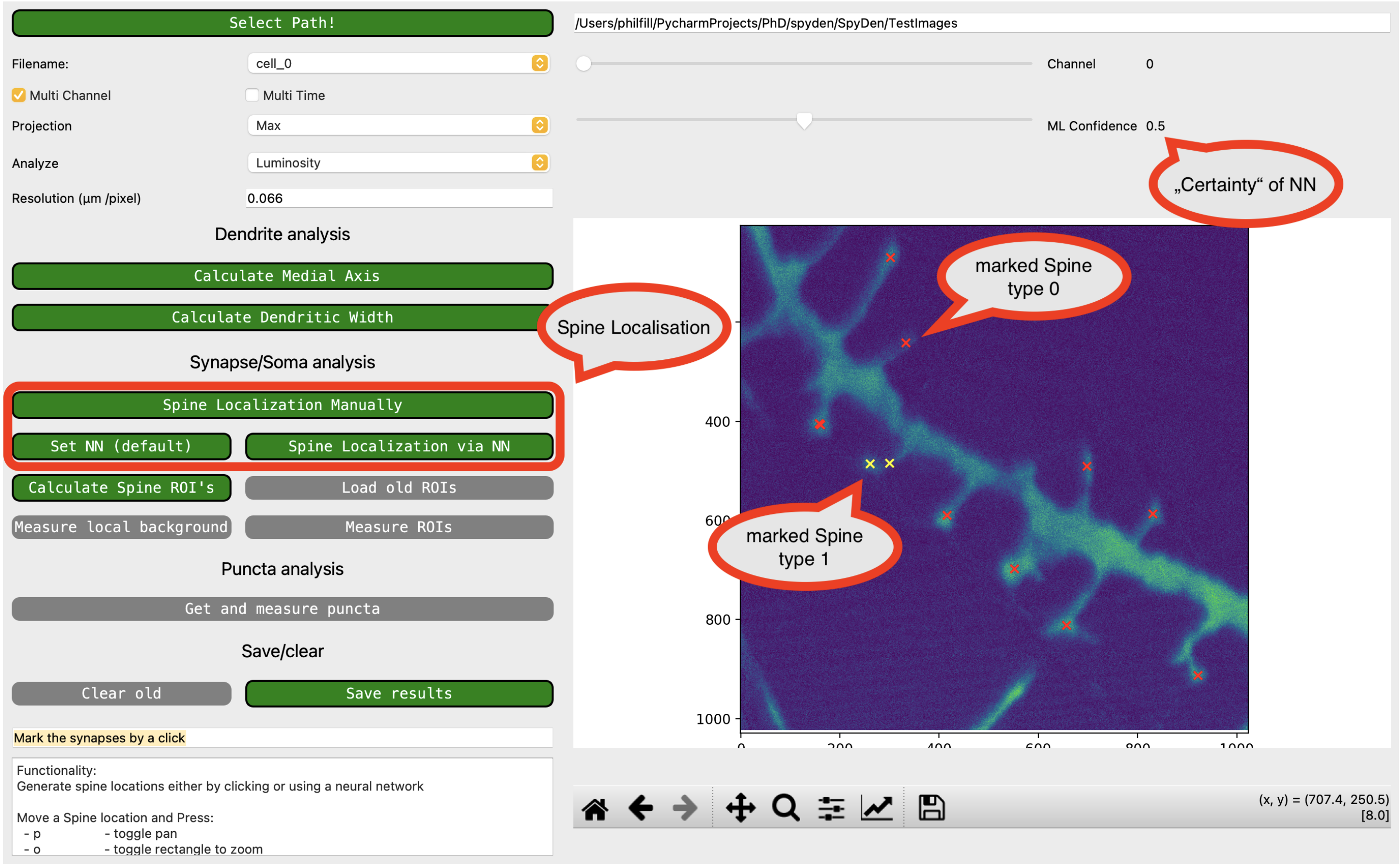


### Handy Shortcuts for Spine Marking

- **Left-click**: Marks a spine head.
- **p**: Toggles between panning mode and mouse interaction.
- **o**: Activates the rectangular zoom tool.
- **h**: Resets the view to the default scale.
- **d**: Deletes a spine head (hover over the point, then press **d**).
- **Backspace**: Deletes all marked spine heads.
- **Ctrl + Left-click**: Changes the classification of a selected spine head (e.g., to another spine type), if multiple classes are defined. Importantly, spine necks will not be calculated with this classification.
- **Shift + Left-click**: Changes the classification of a selected spine head (e.g., to another spine type), if multiple classes are defined.

After marking all relevant spines, you can generate a **Region of Interest (ROI)** for each spine. This step also calculates the **neck length** of each spine: the distance from the edge of the spine head ROI to either the dendritic medial axis or the dendritic edge (if the dendritic segmentation has been performed).

## 4.2 Spine Segmentation

Following the automatic (or manual) localization of each spine head, SpyDen provides a segmented ROI. The software:

- Positions a **circular or polygonal region** around the spine head.
- Identifies boundaries of spine head and neck using edge-detection algorithms (e.g., the Canny Edge Detector).
- Generates a polygonal mesh that you can further refine manually.

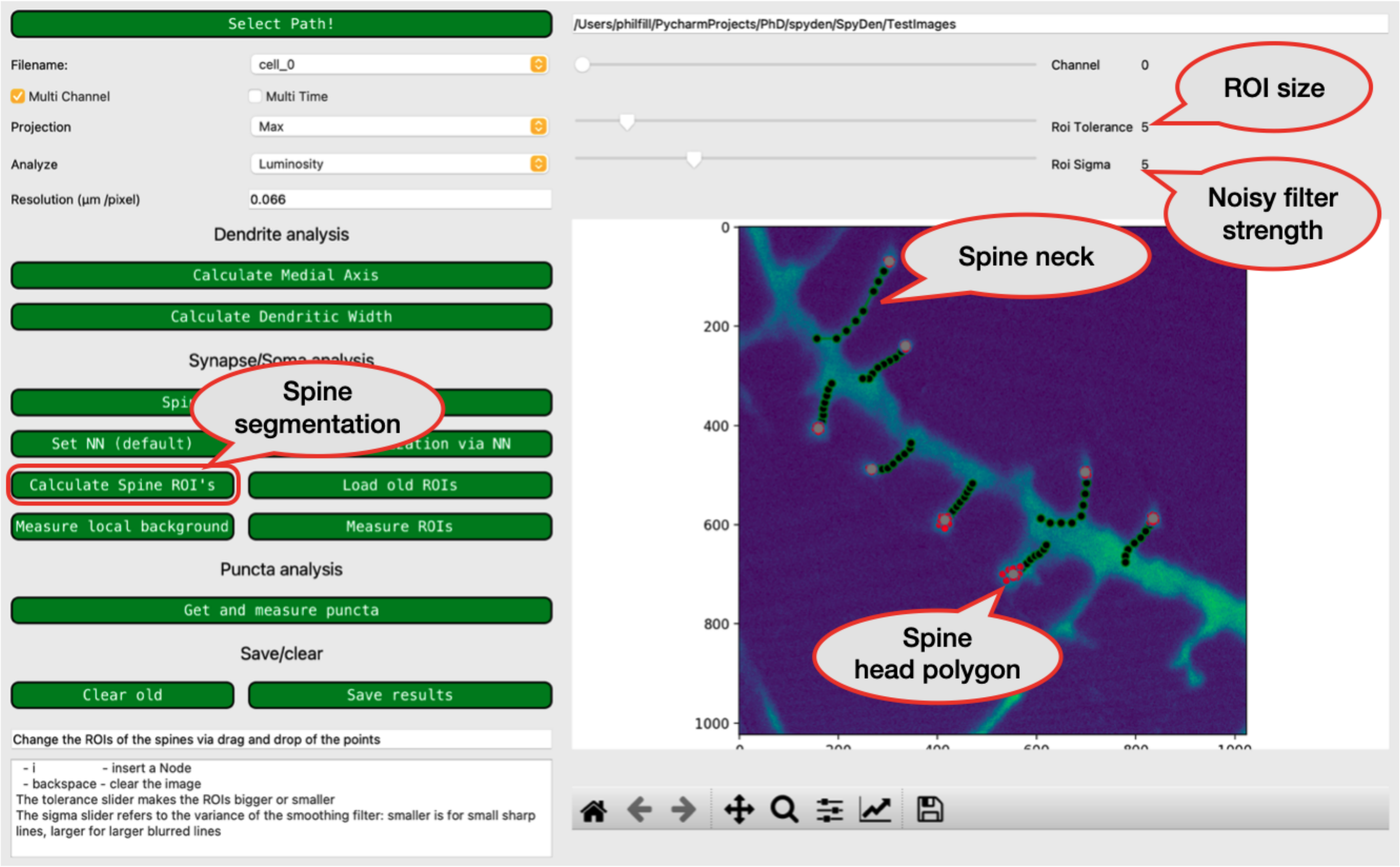


**Additional Shortcuts for Segmentation**
These shortcuts can be used to interact with the individual polygons to enhance the ROI segmentation (see figure on the right).

- **t**: Toggles between draggable polygon vertices and line interaction.
- **i**: Inserts a new vertex (hover over an existing polygon edge, then press **i**).
- **d**: Delete an existing vertex (hover over the vertex, then press **d**).
- **Backspace**: Deletes all spine markings and polygons.
- **Tolerance & Sigma**: Adjust these sliders to configure the Canny Edge Detector for different image conditions (e.g., noise, contrast levels).

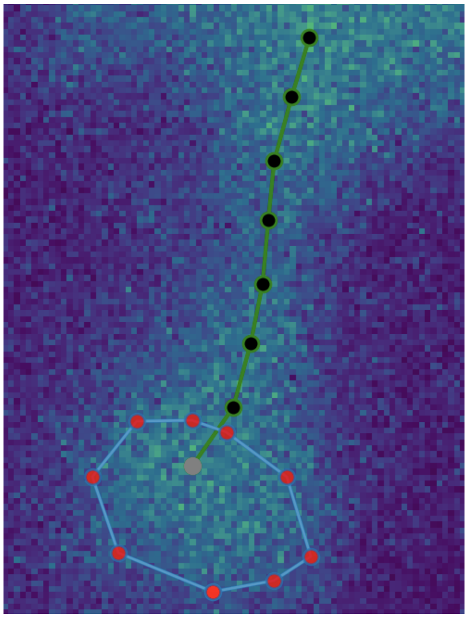

**Recommendation**: Use **zoom** (either via the navigation bar at the bottom or the keyboard shortcuts) to closely inspect small details and fine-tune the polygon points. By default, each spine ROI starts as an octagon (N, E, S, W, NE, SE, SW, NW) centred on the spine head. These points can be moved or supplemented as needed.

## 4.3 Spine neck calculation

As can be seen in the Figure on the left, spine necks can be similarly manually adjusted as the dendritic medial axis path/spine head ROIs. **Importantly**: Spine necks will be calculated from the center of the spine to medial axis path, or **if the dendritic width has been calculated** the edge of the dendrite. The length is then from the edge of the spine head and the end of the spine neck.

The spine neck is calculated once you press the *Measure ROIs* button and will appear as a yellow polygon surrounding the neck. Similar to the dendritic width calculation, sliders will appear that allow you to adjust the local smoothness and width multiplication factor.

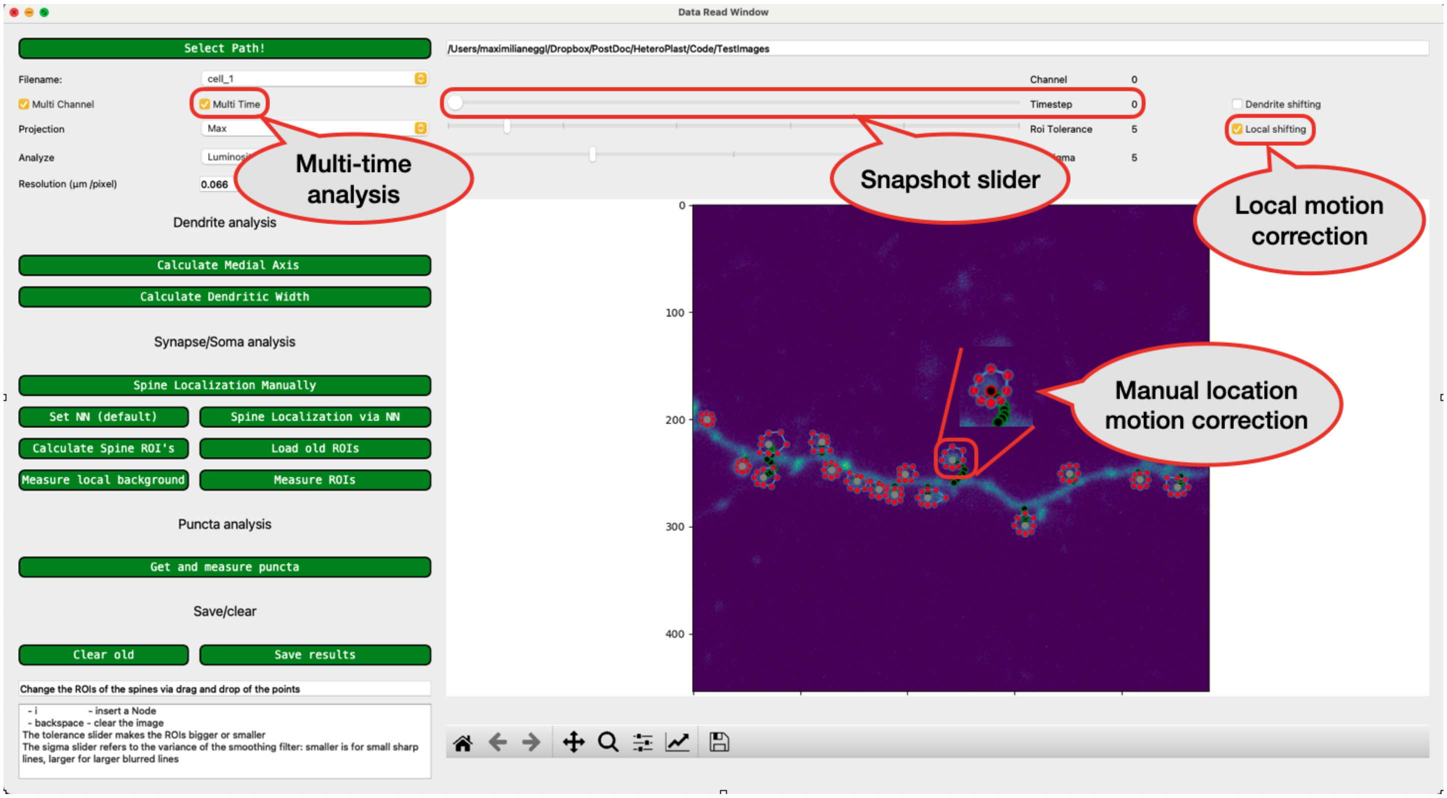


## 4.4 Global and local shifting

### Global correction

In the case that the user is considering data that includes multiple snapshots and wants to study the dynamics of the spines, then they will need to select the multi-time tick-box in the main analysis window. This activates the snapshot slider that allows the user to seamlessly move through snapshots while keeping their ROIs overlaid.

Once multi-time is selected, an **automatic global motion correction** is applied to stabilise the image and ensure that no drift is observed in the ROIs. The values of this correction can be found in the *MinDir.npy* file, and can be amended manually if necessary. We have also included the "Dendritic shifting" option (which at this point is still an experimental feature), where the selected dendritic stretch is used as a reference for the motion correction algorithm (in this case the corrections can be found in *MinDirD.npy*).

### Local correction

However, it is possible that spines also shift locally, a fact that is not considered in the global motion correction. In this case, the user can select the local shifting button which appears once the user has clicked *Calculate Spine ROIs* with the multi-time tickbox selected.

This will recalculate the ROIs with local shifting in mind. Particularly, the user can correct this local shifting in each timestep by clicking the center of the spine (which will be filled black with a red border if active) and dragging it to the new location (see example in the figure above)

## 4.5 Local background calculation

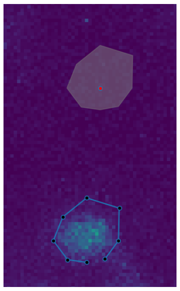

If the user wishes to study the luminosity of spines, it is key that the local background to each spine is also calculated. So, while a general background (consisting of the corners of the images which do not contain any dendritic of spine structures) is calculated and provided in the *background.npy* file, the user also has the possibility of calculating the local background to each spine using the *Measure local background button*. In this mode, the spine head ROIs are taken and projected onto a reasonable location close to the spine. This ROI (which has a gray outline with a red center point - see picture on the left) then serves as the site for the calculation of background metrics.

The user can interact with this ROI by selecting and dragging the red center point if the automatic location is not suitable.

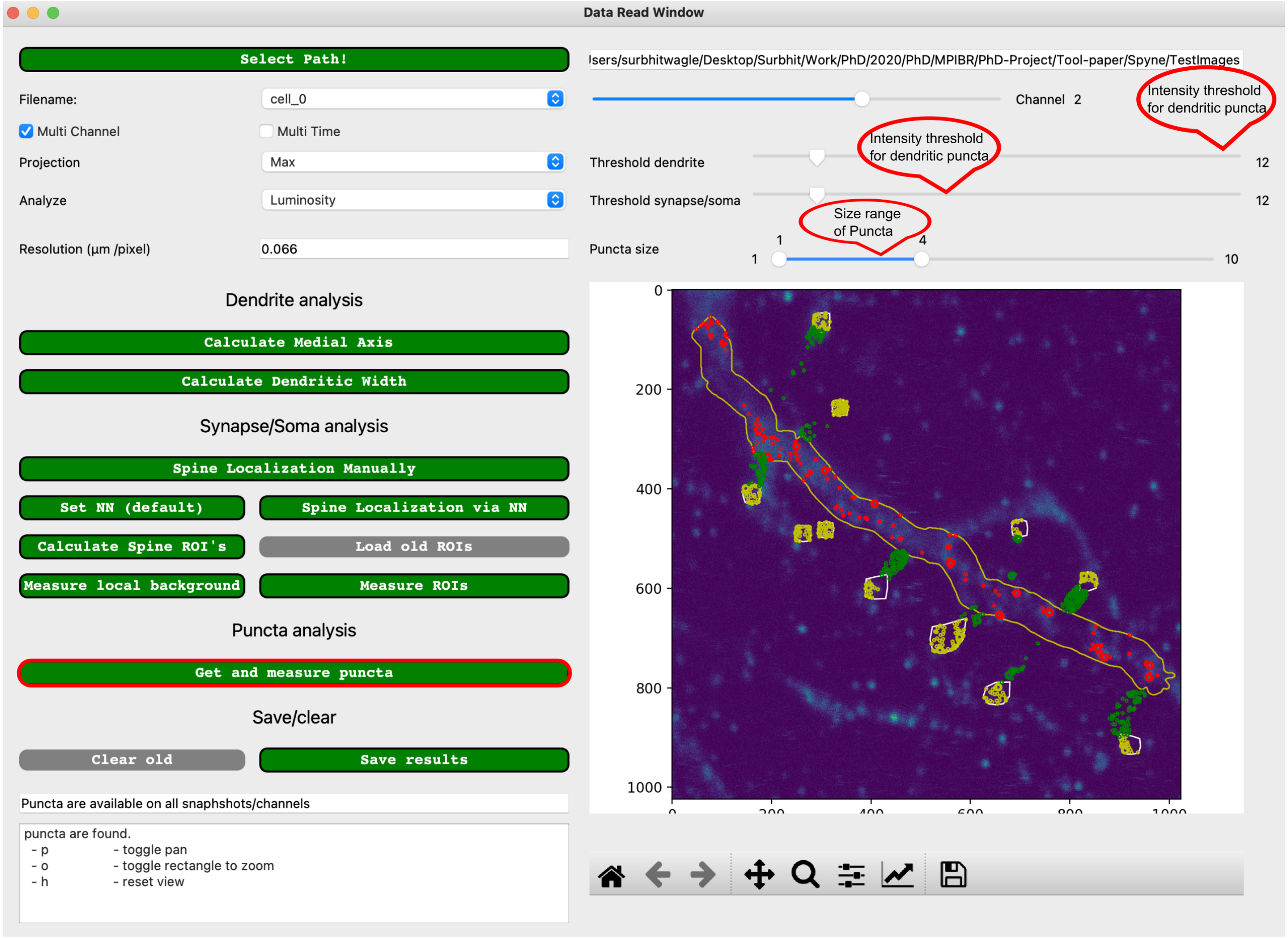


# 5 Puncta detection

SpyDen also allows for the detection and analysis of bright fluorescent puncta on a dark background using the blob_log method that uses the Laplacian of Gaussian underneath for puncta detection. Once ROIs for dendritic, spine, and soma are generated, puncta can be detected in each of the ROIs. SpyDen automatically calculates the important statistics for each puncta and saves them. To use, click on the "Get and measure puncta" button.

The blob detection feature comes with three sliders.

- Threshold dendrite: percentage of maximum intensity value in each dendritic and spine neck ROI as absolute lower bound for scale space maxima. A higher value of threshold would lead to lower number of detected puncta.
- Threshold synapse/soma: percentage of maximum intensity value in each spine and soma ROI as absolute lower bound for scale space maxima. A higher value of threshold would lead to lower number of detected puncta.
- Puncta size: the minimum and maximum standard deviation ($\sigma$) of the Gaussian kernel.

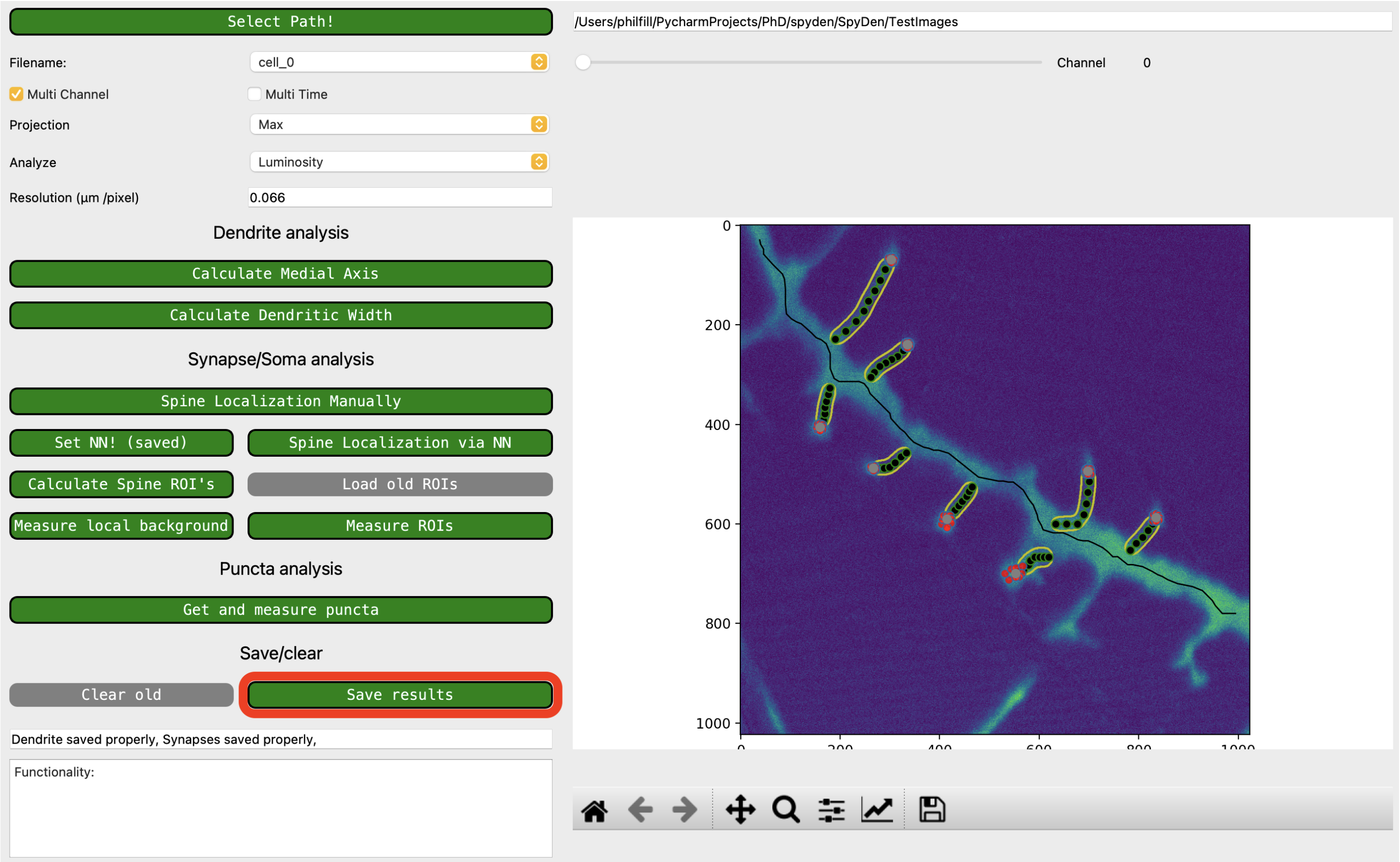


# 6 Output

Once you have completed all desired analyses—dendrite and spine segmentation, puncta detection, etc.—SpyDen displays an overview of the identified structures:

## 6.1 Saving Results

Through the **Save** function, all data are stored, including:

1. **Statistical Evaluations**
   - Integrated intensities (e.g., fluorescence values across channels)
   - Geometrical measurements (e.g., spine neck lengths, mean dendritic width)
   - Additional metadata (sampling intervals, timepoints, applied parameters)
2. **Segmentation Masks**
   - Dendrite masks (e.g., `dendrite_mask.png`)
   - Spine masks (e.g., `spine_mask.png`)
3. **Additional Visualizations** (if enabled)
   - Overlays showing segmentations projected onto the raw image
   - Marked axis paths and polygons for spines

A structured folder hierarchy is created in the target directory, facilitating easy post-processing or archiving of results:

| | | | |
|---|---|---|---|
| cell_0 | Heute, 13:03 | -- | Ordner |
| background.npy | Heute, 13:03 | 160 Byte | Dokument |
| cell_0.tif | Gestern, 15:45 | 16,8 MB | TIFF-Bild |
| Dendrite | Heute, 13:05 | -- | Ordner |
| Dendrite_Channel_0.csv | Heute, 13:03 | 70 KB | CSV-Dokument |
| Dendrite_Channel_1.csv | Heute, 13:03 | 69 KB | CSV-Dokument |
| Dendrite_Channel_2.csv | Heute, 13:03 | 71 KB | CSV-Dokument |
| Dendrite_Channel_3.csv | Heute, 13:03 | 71 KB | CSV-Dokument |
| Dendrite0.npy | Heute, 13:03 | 2 KB | Dokument |
| Dendrites.json | Heute, 13:03 | 2 KB | JSON |
| ImageJ | Heute, 13:03 | -- | Ordner |
| DendMed_0.roi | Heute, 13:03 | 14 KB | Dokument |
| DendSeg_0.roi | Heute, 13:03 | 10 KB | Dokument |
| Mask_dend0.png | Heute, 13:03 | 6 KB | PNG-Bild |
| Settings.txt | Heute, 13:03 | 352 Byte | Reiner Text |
| Spine | Heute, 13:03 | -- | Ordner |
| ImageJ | Heute, 13:03 | -- | Ordner |
| Mask_0.png | Heute, 13:03 | 3 KB | PNG-Bild |
| Mask_1.png | Heute, 13:03 | 3 KB | PNG-Bild |
| Mask_2.png | Heute, 13:03 | 3 KB | PNG-Bild |
| Mask_3.png | Heute, 13:03 | 3 KB | PNG-Bild |
| Mask_4.png | Heute, 13:03 | 3 KB | PNG-Bild |
| Mask_5.png | Heute, 13:03 | 3 KB | PNG-Bild |
| Mask_6.png | Heute, 13:03 | 3 KB | PNG-Bild |
| Mask_7.png | Heute, 13:03 | 3 KB | PNG-Bild |
| ROIs.png | Heute, 13:03 | 324 KB | PNG-Bild |
| Synapse_l_Channel_0.csv | Heute, 13:03 | 3 KB | CSV-Dokument |
| Synapse_l_Channel_1.csv | Heute, 13:03 | 2 KB | CSV-Dokument |
| Synapse_l_Channel_2.csv | Heute, 13:03 | 2 KB | CSV-Dokument |
| Synapse_l_Channel_3.csv | Heute, 13:03 | 2 KB | CSV-Dokument |
| Synapse_l.json | Heute, 13:03 | 30 KB | JSON |
| cell_1 | Heute, 12:50 | -- | Ordner |
